# Comparative genome analysis investigation of nosocomial and community-acquired cases of Legionnaires’ disease caused by ST2858 and ST378

**DOI:** 10.1128/spectrum.00513-25

**Published:** 2025-06-09

**Authors:** Maria Najeeb, Gillian Cameron, Marianne Grimard-Conea, Sara Matthews, Julie Brodeur, Geneviève Cadieux, Pierre A. Pilon, Xavier Marchand-Senécal, Cindy Lalancette, Martin Smith, Michèle Prévost, Sebastien P. Faucher

**Affiliations:** 1 Department of Natural Resource Sciences, McGill University 444590, Sainte-Anne-de-Bellevue, Québec, Canada; 2 Department of Civil, Geological and Mining Engineering, Polytechnique Montréal 467653 , Montréal, Québec, Canada; 3 Direction régionale de santé publique de Montréal, Centre intégré universitaire de santé et de services sociaux du Centre-Sud-de-l’île-de-Montréal 49987, Montréal, Québec, Canada; 4 School of Global and Public Health, McGill University 5620, Montréal, Québec, Canada; 5 École de santé publique, Université de Montréal 248214 , Montréal, Québec, Canada; 6 Department of Microbiology, Infectious Diseases and Immunology, Faculty of Medicine, Université de Montréal 12368 , Montréal, Québec, Canada; 7 Infectious Diseases Division, Department of Medicine, Hôpital Maisonneuve-Rosemont, Centre intégré universitaire de santé et de services sociaux de l’Est-de-l’île-de-Montréal 439499 , Montréal, Québec, Canada; 8 Laboratoire national de santé publique du Québec, Sainte-Anne-de-Bellevue, Québec, Canada; 9 School of Biotechnology and Biomolecular Sciences, UNSW Sydney 98492, Sydney, New South Wales, Australia; 10 Department of Biochemistry and Molecular Medicine, Faculty of Medicine, Université de Montréal 195983 , Montréal, Québec, Canada; 11 CHU Sainte-Justine Research Centre 70443 , Montréal, Québec, Canada; 12 Industrial Chair on Drinking Water, Department of Civil, Geological and Mining Engineering, Polytechnique Montréal 467653 , Montréal, Québec, Canada; 13 Centre de Recherche en Infectiologie Porcine et Avicole (CRIPA), Faculté de Médecine Vétérinaire, Université de Montréal 70354 , Saint-Hyacinthe, Québec, Canada; 14 Centreau – Centre québécois de recherche sur la gestion de l’eau, Université Laval 4440, Québec City, Québec, Canada; Michigan State University, East Lansing, Michigan, USA

**Keywords:** *Legionella pneumophila*, co-infection, copper resistance, hot water distribution systems, single nucleotide polymorphism, healthcare facility

## Abstract

Eight cases of Legionnaires’ disease caused by *Legionella pneumophila* serogroup 1 sequence type (ST) ST2858 were detected within 4 years in Montréal, Canada. Most cases were associated with a single healthcare facility, and one of them presented with a co-infection, from which ST378 was isolated as well. The source of ST2858 was not identified, despite extensive environmental sampling. The goal of this study was to determine the diversity of both STs and confirm the source of each by matching clinical to environmental isolates. Comparative genome analysis was performed to investigate the genetic relatedness of the isolates. Long- and short-read hybrid assembly was used to produce high-quality closed genomes. A PCR assay, amplifying a unique gene of ST2858, was also developed. None of the 599 environmental isolates screened by PCR was ST2858. The ST2858 clinical isolates had a maximum of two single nucleotide polymorphisms in pairwise comparison, suggesting a common source. ST378 isolates had higher genomic diversity, and the isolate from the co-infection was similar to environmental ST378 from the healthcare facility’s hot water distribution system. The isolate from the co-infection harbored a plasmid conferring increased copper resistance. Understanding genomic diversity could help identify potential sources and should be considered for matching clinical with environmental isolates. Phenotypic diversity may be relevant for outbreak investigation, surveillance, and management.

**IMPORTANCE:**

Legionnaires’ disease (LD) is transmitted to humans by inhalation of aerosols contaminated with *Legionella*. When an outbreak occurs, identification of the source allows public officials to make sure the source is controlled to prevent further cases. In this study, whole genome sequencing was used to investigate the relatedness between clinical and environmental isolates collected during the epidemiological investigation of cases of LD centered around a single healthcare facility, providing valuable information about the diversity of *Legionella* within water systems and similarity thresholds for matching clinical and environmental strains. The genomic data were also used to design a methodology to rapidly screen hundreds of historical isolates and DNA extracts, which could benefit source identification in other outbreaks. Furthermore, *Legionella* isolates may differ in their ability to resist disinfection methods and potentially acquire novel genetic determinants, and water system characteristics may select for specific *Legionella* strains.

## INTRODUCTION

*Legionella* are water-borne opportunistic pathogens responsible for Legionnaires’ disease (LD), a severe form of pneumonia in humans (1). About half of the 73 species of *Legionella* have been reported to cause disease in humans (2); however, *Legionella pneumophila* serogroup 1 is responsible for ~80% of the cases for which an isolate can be typed (3). The bacterium inhabits water systems while relying on protozoans, including amoeba and ciliates, for replication (1). Engineered water systems (EWS) such as water cooling towers (WCTs), whirlpools, and hot water distribution systems (HWDS) can harbor *L. pneumophila*, leading to human infection through aerosolization of the bacterium (4). In humans, *L. pneumophila* infects alveolar macrophages and proliferates intracellularly using the Icm/Dot Type IVb secretion system to translocate effector proteins into host cells (5).

Recent estimates by the US Centers for Disease Control and Prevention (CDC) highlight the increasing burden of water-borne opportunistic pathogens, such as *L. pneumophila*, *Pseudomonas*, and non-tuberculous *Mycobacteria*, with 10,800 hospitalization stays per year and a cost of 402 M$ attributed to *Legionella* in the United States (6–8). LD has a high individual and population burden, representing 3.75% of total DALYs in Europe prior to COVID-19 (9). These pathogens are responsible for the majority of hospitalizations, costs, and fatalities associated with drinking water outbreaks (8). According to the Canadian Notifiable Disease Surveillance System, the number of LD cases in Canada has increased approximately 15-fold between 2004 and 2019 (Public Health Agency of Canada (10). Worldwide increase in the prevalence of LD is likely due to aging populations and infrastructures, improved surveillance, urbanization, and climate change, but the contribution of each of these factors is uncertain (11). In Quebec, LD became a notifiable disease in 1987 and shows increasing incidence since 2006 (12). In 2012, Quebec City reported an outbreak consisting of 183 cases and 13 deaths (13). As a result, the Quebec government enacted regulations for the operation and maintenance of WCTs (14).

Confirming the source of LD outbreaks requires matching *L. pneumophila* clinical strains with environmental strains. This allows public health officials to ensure that the source has been eliminated or controlled, thus preventing further cases and protecting the community from continued exposure (15). Confirming the source also enables targeted remediation efforts, such as disinfection of water systems, cleaning WCTs, or replacing contaminated plumbing fixtures (16). Additionally, it aids in understanding the bacteria’s transmission pathways and the environmental conditions favoring its growth (17). Moreover, identifying and addressing the outbreak’s source helps prevent future occurrences by improving maintenance and surveillance protocols and possibly updating regulations (17)^18^.

Sequence-based typing (SBT), consisting of analyzing the sequence of specific regions of seven housekeeping genes (*flaA*, *pilE*, *asd*, *mip*, *mompS*, *proA*, and *neuA*), is used to classify strains into sequence types (STs) (19). While SBT has been instrumental in identifying strains, it falls short in investigating complex outbreaks involving closely related strains of the same ST, which requires more discriminating techniques such as whole-genome sequencing (WGS) for higher resolution (20). WGS is now considered the gold standard to investigate and confirm the source of LD outbreaks (13, 21–25). This approach enables precise genetic characterization of strains involved in outbreaks. For instance, several outbreak investigations have been performed using this technique to confirm suspected environmental sources, including WCTs and hot water systems in hospital-associated outbreaks (26, 27). Moreover, this approach facilitated the comprehensive investigation of genetic diversity among isolates responsible for various outbreaks over multiple years, enabling the identification of specific genetic factors contributing to this diversity (21). A study focusing on the investigation of legionellosis in New York State highlighted that WGS can improve strain differentiation compared to that of pulsed-field-gel electrophoresis or SBT, suggesting a crucial role for WGS in laboratory investigations of LD outbreaks (24).

In the summer of 2019, a mixed community and nosocomial outbreak of legionellosis was reported in a small area in Montréal, consisting of 14 cases (28). All cases were determined to be caused by *L. pneumophila* serogroup 1 based on urinary antigen tests or serotyping of isolates. Based on the incubation period of LD and the cases’ locations, two subclusters of LD cases were identified: four cases were associated with an acute care healthcare facility (HF) (hereafter referred to as HF-A) and three cases lived in the same retirement home nearby. Isolates could be retrieved from 5 of the 14 cases, including 2 from HF-A cases, 1 from the retirement home cases, 1 from a cancer care home, and 1 case whose exposure setting was most likely a private home nearby. One isolate showed a complete ST2858 SBT profile; partial SBT profiles were obtained for the other four isolates, suggesting they were also ST2858. No matching environmental source was found despite sampling and analyzing 30 WCTs, as well as the HF-A and retirement home water systems (28). In 2021, another outbreak investigation in the same area of Montréal revealed three more cases infected by ST2858, with one of those cases showing a co-infection with ST2858 and ST378. This case and another one were associated with HF-A, while the third one was associated with a retirement home (different from the one in 2019). Epidemiological investigations suggested a common source, located in proximity to HF-A, but extensive sampling in 2021 also failed to identify an environmental source for ST2858, indicating potential minor or hidden sources. A retrospective investigation revealed that a nosocomial case from 2018 was also infected by ST2858. This also raises the question of whether the ST2858 isolates from 2018, 2019, and 2021 are clonal; if not, multiple sources might have caused these cases. In contrast, ST378 has been historically found in various water systems in Montréal, but the source of ST378 in the case of the co-infection was not confirmed, although the HWDS of HF-A was suspected. The objectives of this study were to: (i) determine the diversity of ST2858 clinical isolates from three different years to evaluate the likelihood of a common source, (ii) identify the most likely source of ST378 from the 2021 case with the co-infection suspected to be the water system of HF-A, and (iii) evaluate the diversity of ST378 from different LD cases and environmental systems since 2007.

## RESULTS

### WGS of clinical and environmental isolates of *L. pneumophila*

In total, 67 isolates were subjected to WGS in this study and are described in detail in Table 1; Table S1. Ten of these were clinical ST2858 isolates, including one from 2018, four from 2019, and five from 2021 (including two isolates, SPF582 and SPF583, from the co-infection case and two isolates from another case, SPF585 and SPF586). The ST378 isolates included in this analysis come from 8 clinical samples (including SPF581, from the co-infection in 2021) and 43 environmental samples, recovered between 2007 and 2021 from various healthcare facilities and their associated HWDS.

**TABLE 1:** Summary of the isolates selected for this study along with their source, type, ST and, if available SG^*a*^

Strain ID	Type^*b*^ (case number)	Source or exposure setting^*c*^	Year of isolation	ST	SG
SPF544	E	HF-A	2021	ST378	Lp10
SPF580	C (8)	Retirement home A^*d*^	2021	ST2858	Lp1
SPF581	C (12)	HF-A	2021	ST378	Lp4/10
SPF582	C (12)	HF-A	2021	ST2858	Lp1
SPF583	C (12)	HF-A	2021	ST2858	Lp1
SPF584	C (14)	Private home^*e*^	2019	ST2858	Lp1
SPF585	C (19)	HF-A	2021	ST2858	Lp1
SPF586	C (19)	HF-A	2021	ST2858	Lp1
SPF587	C (23)	Cancer care home^*f*^	2019	ST2858	Lp1
SPF588	C (24)	HF-A	2019	ST2858	Lp1
SPF589	C (26)	Retirement home B^*g*^	2019	ST2858	Lp1
SPF590	C (13)	HF-A	2018	ST2858	Lp1
SPF597	E	HF-A	2021	ST378	Lp10
SPF599	E	HF-A	2021	ST378	Lp10
SPF600	E	HF-A	2021	ST378	Lp10
SPF601	E	HF-A	2021	ST378	Lp10
SPF629	E	HF-A	2015	ST378	Lp9
SPF630	E	HF-A	2015	ST378	Lp10
SPF631	C	HF-A	2020	ST378	Lp10
SPF633	E	HF-A	2015	ST378	Lp10
SPF634	E	HF-A	2015	ST378	Lp9
SPF635	E	HF-B	2017	ST378	Lp10
SPF636	E	HF-B	2017	ST378	Lp10
SPF637	C	HF-A	2007	ST378	Lp10
SPF638	C	HF-A	2008	ST378	Lp10
SPF639	C	HF-C	2009	ST378	Lp10
SPF640	E	HF-C	2011	ST378	Lp10
SPF641	E	HF-C	2011	ST378	Lp10
SPF642	E	HF-C	2011	ST378	Lp10
SPF643	E	HF-C	2011	ST378	Lp10
SPF644	C	HF-C	2011	ST378	Lp10
SPF645	E	HF-C	2011	ST378	Lp10
SPF646	E	HF-C	2011	ST378	Lp10
SPF647	C	HF-C	2011	ST378	Lp10
SPF648	E	HF-C	2011	ST378	Lp10
SPF649	E	HF-C	2011	ST378	Lp10
SPF650	E	HF-C	2011	ST378	Lp10
SPF651	E	HF-C	2012	ST378	Lp10
SPF652	E	HF-C	2012	ST378	Lp10
SPF653	E	HF-C	2012	ST378	Lp10
SPF654	E	HF-C	2012	ST378	Lp10
SPF655	E	HF-C	2012	ST378	Lp10
SPF656	E	HF-C	2012	ST378	Lp10
SPF657	E	HF-C	2012	ST378	Lp10
SPF658	E	HF-C	2012	ST378	Lp10
SPF659	C	HF-D	2012	ST378	Lp10
SPF660	E	HF-C	2012	ST378	Lp10
SPF661	E	HF-C	2013	ST378	Lp10
SPF664	E	HF-A	2015	ST378	Lp10
SPF665	E	HF-A	2015	ST378	Lp10
SPF666	E	HF-A	2015	ST378	Lp10
M34295	E	HF-A	2021	ST378	Lp10
SPF938	E	HF-A	2022	ST378	ND
SPF939	E	HF-A	2022	ST378	ND
SPF940	E	HF-A	2022	ST378	ND
SPF941	E	HF-A	2022	ST378	ND
SPF942	E	HF-A	2022	ST378	ND
SPF943	E	HF-A	2022	ST378	ND
SPF944	E	HF-A	2022	ST378	ND
SPF945	E	HF-A	2022	ST378	ND
SPF947	E	HF-A	2022	ST378	ND

^*a*^ ND: not determined.

^*b*^ Type of isolates: E, environmental; C, clinical.

^*c*^ For clinical isolates the most probable exposure setting based on epidemiological investigation is indicated. HF: healthcare facility.

^*d*^ Visited HF-A 16 days prior to the start of the symptoms. This retirement home is nearby HF-A.

^*e*^ Visited HF-A 38 days prior to the start of the symptoms. This private home is nearby HF-A.

^*f*^ Visited HF-A 6 days prior to the start of the symptoms for only 2 h. The cancer care home is nearby HF-A.

^*g*^ Never visited HF-A. This retirement home is nearby HF-A.

For all ST2858 (*n* = 10), short and long reads were combined to obtain closed genomes. Presumed ST2858 isolates, due to incomplete SBT profiles, were confirmed to be ST2858 by WGS. All isolates were presented with a single chromosome averaging 3.4 Mb with no plasmid. Hybrid assemblies were also generated for 33 of the ST378 isolates, including 1 clinical isolate from the 2021 co-infection, 6 clinical isolates, and 10 environmental isolates from HF-A and 16 from HF-C (Table S2). The rest of the isolates were subjected to short-read sequencing only and assembled using SPAdes. The average size of the ST378 genomes was 3.4 Mb. A plasmid was detected in SPF581. Overall, the N50 value of these genomes ranged from 1.3 to 3.4 Mbp (for the closed genome from hybrid assembly). A general overview of assemblies for each isolate is provided in Table S2. Functional annotation was performed with Prokka showing an average of 2951 coding sequences, 9 rRNA, and 43 tRNA (Table S3). Overall, the assemblies were not substantially contaminated, ranging from 0.9 to 1.9, with 100% completeness as determined by MiGA. The ST of all isolates was confirmed by using legsta.

### Phylogenetic analysis

Phylogenetic analysis of isolates of both STs was performed with kSNP4. ST2858 and ST378 clustered separately and differed by approximately 35,517 single nucleotide polymorphisms (SNPs), suggesting a significant genetic divergence between them. Pangenome analysis showed a similar distinction between both STs, showing the presence of 511 unique genes in ST2858 and 765 unique genes in ST378. The majority of unique genes in ST2858 were annotated as hypothetical proteins; those with putative function included protein TraK, translational regulator CsrA, prophage integrase IntS, and CRISPR-associated proteins and tyrosine recombinase XerC, among others (Fig. S1; Table S4).

**Fig 1:**
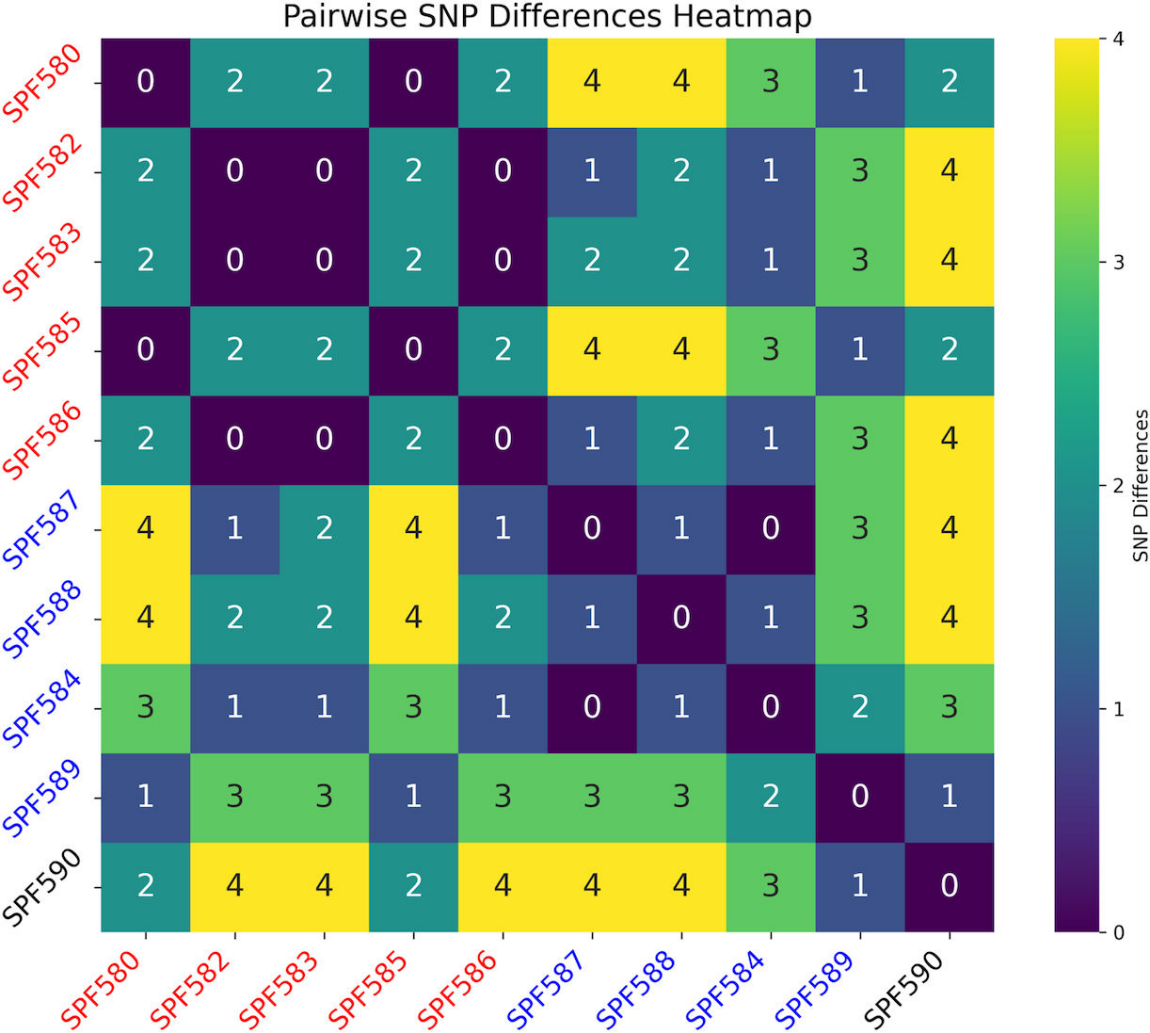
Whole-genome-based SNP analysis of the 10 ST2858 isolates. Core SNP analysis was conducted using kSNP4, and the resulting pairwise comparison matrix is shown. The isolates are colored according to the year of isolation: red, 2021; blue, 2019; and black, 2018.

SNP-based phylogenetic analysis of the 10 ST2858 clinical isolates alone revealed that they are highly clonal (Fig. 1). No recombination events were detected using Gubbins (data not shown), which uses high SNP density as a marker for recombined regions. The maximum number of SNPs detected between any pair of the isolates was only 4. These results, in combination with a few genomic reorganizations revealed by Mauve (Fig. S2), suggest very low diversity between the ST2858 isolates, providing evidence toward a common source of infection over the years. DefenseFinder (29–31) identified several antiphage systems present in all ST2858 genomes including CRISPR-Cas, Restriction-Modification (RM), and Abortive Infection (Abi) systems. The presence of these systems provides support for the clonality of ST2858 isolates and apparent absence of genetic changes over time.

**Fig 2:**
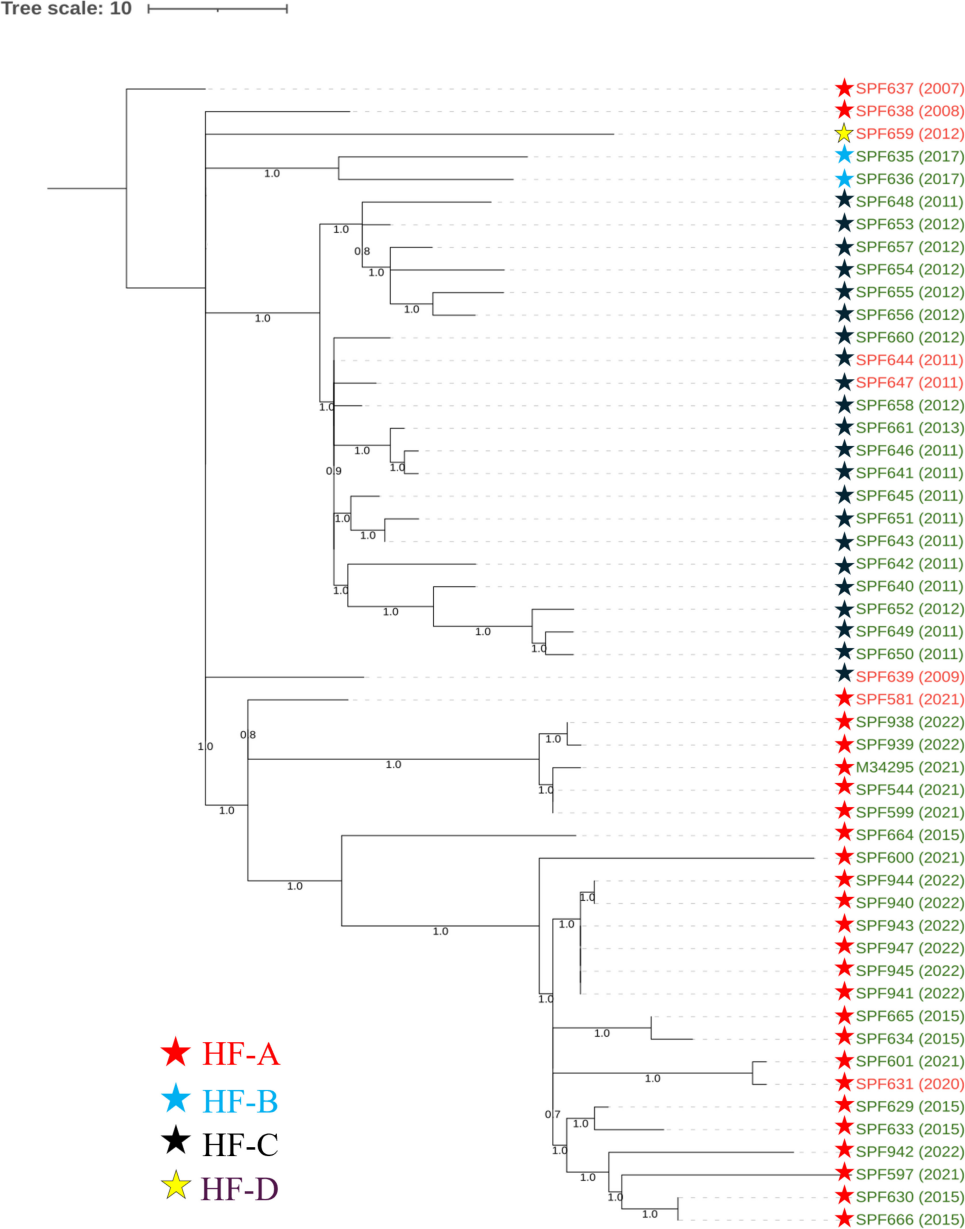
Phylogeny of environmental and clinical ST378 isolates. A maximum likelihood (ML) tree was constructed using SNPs falling outside recombination regions using Gubbins with the --tree-builder option set to *IQ-TREE*. Branch support was evaluated using 1,000 bootstrap, and transfer bootstrap support was enabled (--transfer-bootstrap: TRUE) to provide higher support values for phylogenetic branches. In the final tree, visualized using iTOL, only bootstrap values greater than 70% (0.7) are displayed. The scale bar indicates the number of SNPs per site. Taxa are annotated with colored stars corresponding to their source of isolation, as indicated in the legend. The scale bar at the top represents the number of SNPs per site.

ST378 isolates were obtained from both clinical and environmental samples between 2007 and 2022 from four different healthcare facilities all located in Montréal (HF-A, HF-B, HF-C, and HF-D). Based on kSNP4 analysis, 645 core SNPs were detected across all the genomes of ST378. However, further analysis with Gubbins, using the 2007 clinical isolate SPF637 from a case associated with HF-A as the reference genome, showed that ~38% SNPs were acquired by recombination. The phylogenetic tree, based on polymorphic sites (*n* = 365) outside recombination regions (Fig. 2), indicates that the environmental isolates cluster according to their source of isolation, thereby suggesting probable sources of infection for clinical cases. The three strains that are poorly resolved (SPF638, SPF639, and SPF637) are clinical isolates collected more than 8 years prior to the environmental sampling of HF-A. SPF581 and ST378 isolates from the co-infection clustered with isolates from the HWDS of HF-A, in agreement with the acquisition of infection from within HF-A. Furthermore, most isolates are grouped by their year of isolation, with the maximum pairwise difference recorded between isolates being 73. In addition to the 2021 co-infected case, one other nosocomial ST378 case from 2020 was likely infected by the HWDS of HF-A; the isolate SPF631 clustered with environmental isolates from HF-A. The ST378 isolates showed much more diversity between them than the ST2858 clinical isolates. For example, the ST378 isolate from the 2021 co-infection and an isolate from the 2020 case associated with HF-A showed 37 SNPs (Fig. S3).

One recombination event detected in the ST378 genomes is worth mentioning. It is shared by the two isolates from HF-B, SPF635 and SPF636, containing 233 SNPs over 13 Kb with a maximum recombination-to-mutation (r/m) ratio of 5.97, indicating that the proportion of SNPs derived from recombination events is higher than vertically inherited. Details of recombination location and content are provided in Table S5. Genes affected by recombination events were identified by comparing them to the annotated reference genome.

### PCR screening of isolates and DNA samples for identification of ST2858 sources

Primers were designed to amplify fragments from unique genes harbored by ST2858. Primers designed for genes 786 and 1045 did not produce a fragment from ST2858 and were therefore rejected (Fig. S4). Of the remaining four targets, primers for genes 783, 784, and 3056 amplified a fragment in the non-target isolates ST378, ST1427, and ST213, respectively (Fig. S4 and S5). Primers for gene 787 were the most specific for ST2858 and were chosen for screening of historical isolates and DNA extracts. DNA quality of those samples was confirmed by amplifying the *mip* gene (example shown in Fig. S6). A total of 599 environmental isolates collected in Quebec between September 2020 and December 2022 were screened using ST2858 specific primers, but none were positive for the target gene. Similarly, none of the 52 DNA samples tested were positive.

### ST378 isolates contain a variety of mobile genetic elements

**Fig 3:**
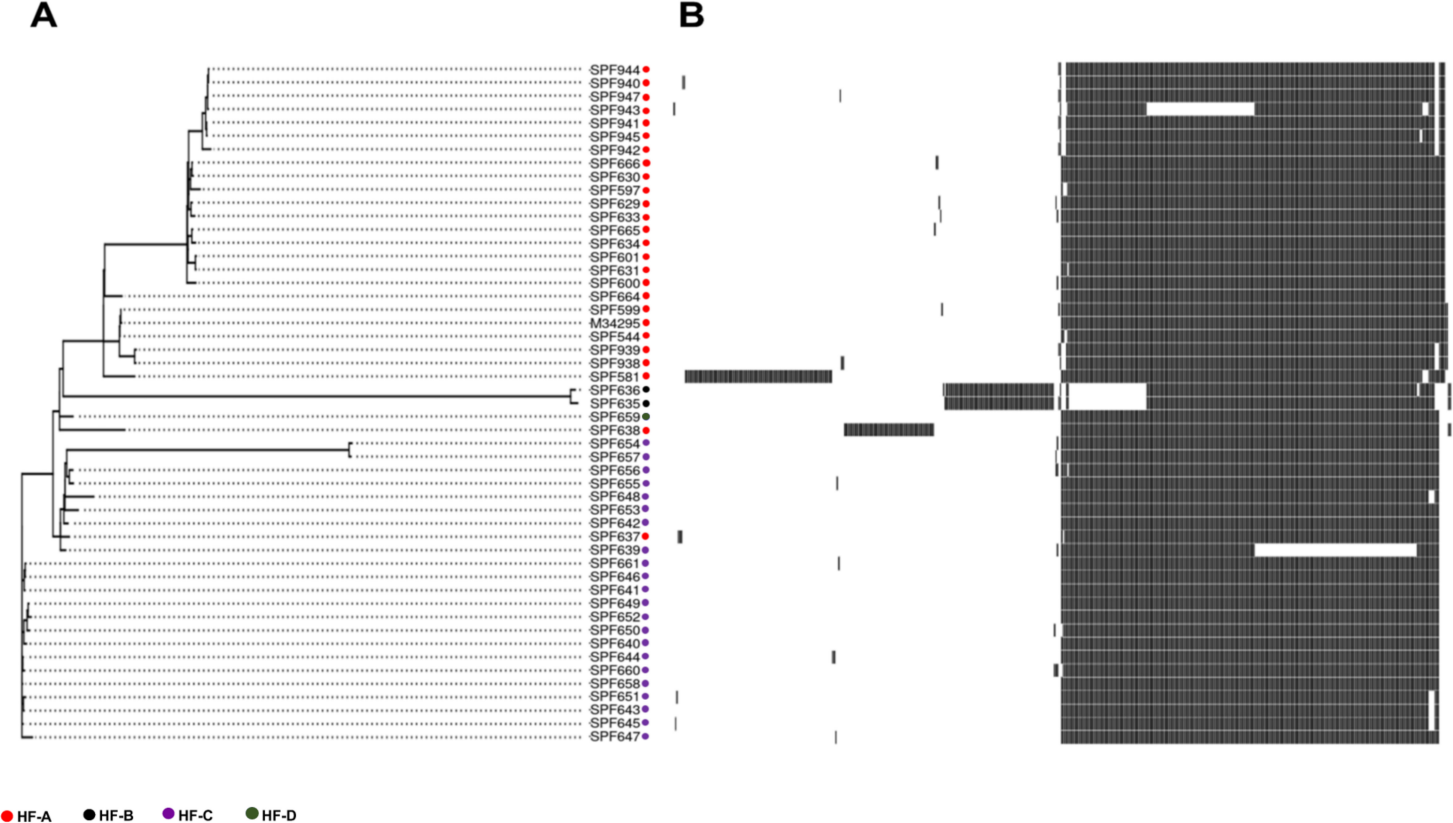
The pangenome analysis of ST378 isolates (*n* = 51) included in the study was performed using Panaroo. (**A**) Core-gene-based phylogeny of ST378 clinical and environmental isolates. Isolates are marked with different colors corresponding to the source (healthcare facility [HF]). (**B**) Matrix showing the variable genes presence (black) and absence (white) in the isolates.

**Fig 4:**
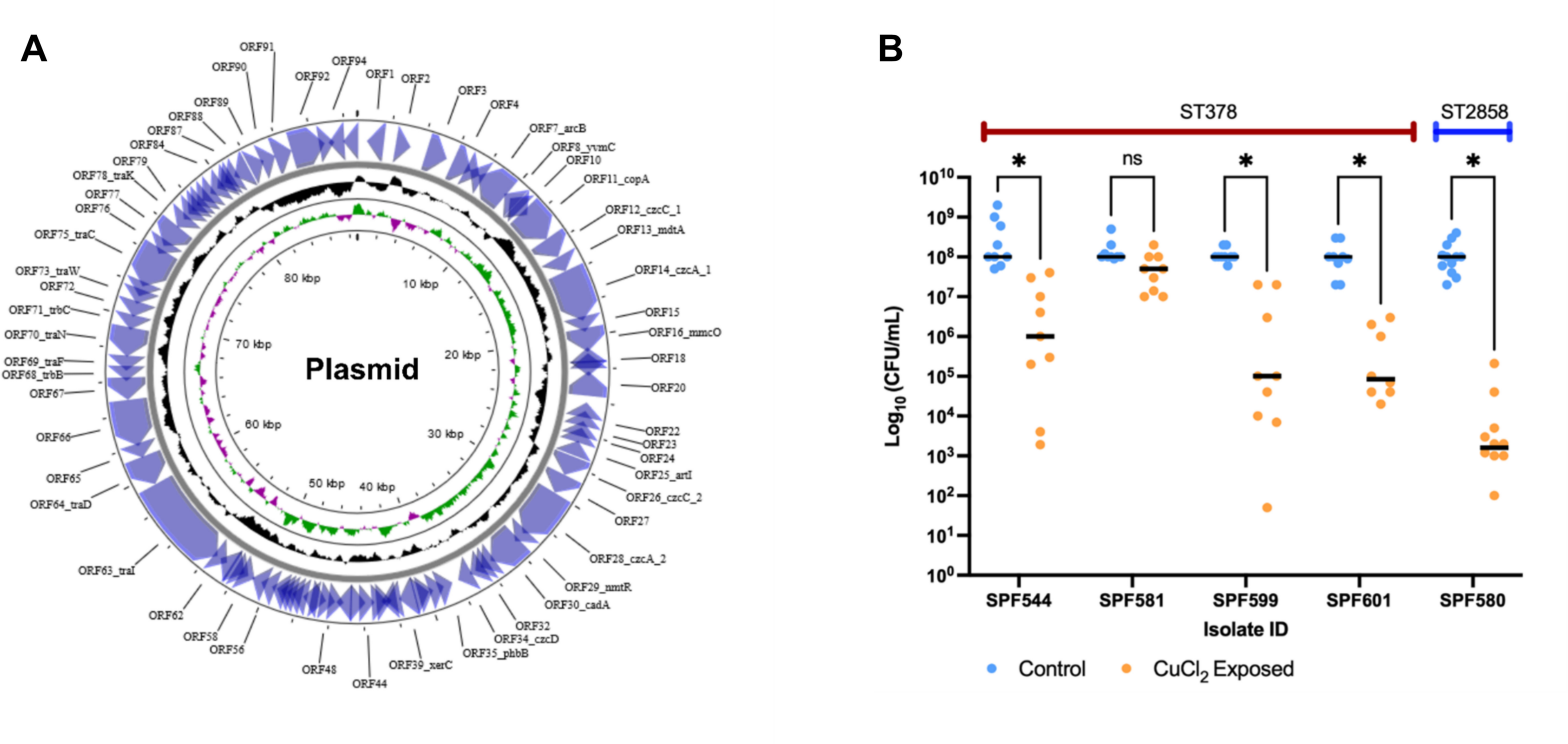
(**A**) Circular map of plasmid detected in SPF581 (ST378) recovered from co-infection. The map was visualized with Proksee. The rings from outside to inside denote coding sequences annotated by Prokka (ring 1, blue), GC content (ring 2, black), and GC skew (ring 3; green, positive; purple, negative). (**B**) Survival of ST378 isolates in the presence of copper. Isolates were grown overnight in AYE and adjusted to Fraquil for 24 h. In triplicate, they were exposed to 0 (control) or 0.08 mM CuCl_2_ (CuCl_2_ Exposed) for 4 h, and a CFU count was used to determine survival in the presence of copper. The experiment was repeated three times with three technical replicates each, and the median of each condition is indicated by the black bar. Unpaired, non-parametric *t*-tests were performed were performed to determine the significance of the effect of copper (ns, non-significant, *P < 0.01).

Pangenome analysis of the 51 ST378 isolates revealed a total of 3,218 genes, comprising 2,699 core genes and 519 accessory genes, including 16 shell (genes shared by ≥15% but <95% of strains), 255 cloud (genes shared by <15% of strains), and 241 soft core genes (genes present in ≥95% but not all strains). Pangenome analysis revealed unique regions in four strains consisting of genes absent in all other genomes (Fig. 3; Table S6). Two were environmental strains SPF635 and SPF636, collected from a different location than all other strains (HF-B). These two strains clustered together and shared the same unique genes in their genome, including those encoded for conjugation machinery and repair mechanisms. Another clinical strain, SPF638, which was also collected from HF-A, harbored a unique chromosomal region mainly containing genes encoding conjugal transfer proteins, Type IV secretion system protein virB4, copper-exporting P-type ATPase, multicopper oxidase MmcO, and other multidrug resistance proteins. Finally, SPF581, the ST378 responsible for the co-infected case, harbored unique genes. The complete, hybrid-assembled genome of that isolate indicates the presence of an 89 kbp plasmid. We estimated that cells of SPF581 carried an average of three copies of this plasmid, based on sequencing depth. Genes encoding for the conjugative transfer system were identified, suggesting the plasmid was most probably acquired through horizontal gene transfer (HGT) (Table S7; Fig. 4A [32]). Analysis against the Comprehensive Antibiotic Resistance Database (CARD) with strict criteria identified no antibiotic resistance genes on the plasmid. To confirm the presence of plasmid-derived sequences, the assembly was checked using BLASTn against the NCBI non-redundant (nr) database. The genome sequences exhibiting (97% or above) homology with our plasmid sequence were used as reference for functional annotation to further confirm the sequence as plasmid. However, genes involved in copper resistance were carried by the plasmid: *copA and mmcO* encoding Copper-exporting P-type ATPase and copper monooxygenase, respectively. Genes involved in cobalt and cadmium resistance were also identified. We hypothesized that SPF581 harboring this plasmid may be more resistant to copper than the other contemporary isolates. Copper susceptibility was tested by exposing ST378 isolates to 0.08 mM CuCl_2_ for 4 h and their survival was monitored by CFU count (Fig. 4B). As expected, SPF581 was not significantly affected by CuCl,_2_ but the survival of the other ST378, SPF544, SPF599, and SPF601, as well as the ST2858 isolate SPF580, was significantly reduced by 0.08 mM CuCl_2_. The CFU count of SPF581 after exposure to copper was significantly higher than all the other strains tested (*P* < 0.05).

### Detection of antimicrobial-resistant (AMR) genes in the isolates

All genomes were also screened for AMR genes against the CARD. Only one resistant gene named *APH (9)-la* was identified in all isolates. This gene is a chromosomal-encoded aminoglycoside phosphotransferase and has been previously found in many *L. pneumophila* isolates (33).

## DISCUSSION

The complete genome sequence of the novel ST2858 isolates associated with two small outbreaks in 2019 and 2021 was successfully obtained. Comparative analysis of hybrid *de novo* assemblies of ST2858 genomes from the 2019 and 2021 outbreaks, as well as the single case from 2018, showed that the isolates are highly homologous, with SNP analysis showing very little diversity with no recombination detected and identical gene content (3,051 core genes out of 3,052 genes with the one extra gene encoding an IS transposase). The presence of various phage defense systems in the genomes of ST2858 could prevent the introduction of new genes and help maintain the clonal nature of the ST2858 isolates. Our results indicate that the same clonal strain caused LD infections from 2018 to 2021 without undergoing major genome changes for at least 3 years. Previous studies suggested an evolution rate for the non-recombinant portion of the genome of 0.35 SNPs/genome/year for ST578 (34) and 0.71 for ST37 (35); however, it is unclear if these figures are conserved between STs, and how this affects population diversity of specific *Lp* lineages in EWS. Our results suggest that the ST2858 population causing these cases underwent a lower substitution rate, possibly due to low environmental pressure, consistent with their presence in a single water system. This is reminiscent of outbreaks of ST23 in Bresso, where the clinical isolates from the 2014 and 2018 outbreaks also showed very similar genomes (36). The 2018 Bresso outbreak consisted of 52 cases, but isolates could only be retrieved from 4 cases. The source of the 2018 outbreak was identified to be a decorative fountain (37). Most environmental isolates from the fountain showed no difference (non-recombinant SNP) with the clinical strains of both the 2014 and 2018 outbreaks (36).

Despite extensive environmental sampling efforts in 2019 and 2021 and screening of 599 environmental isolates and 52 DNA extracts by PCR, the source of ST2858 could not be identified. Failure to match clinical strain with environmental strain is not uncommon, especially for sporadic cases, but it is more successful for large outbreaks (38–40). There are three possibilities for this outcome: (i) the environmental source of the outbreak was not sampled, (ii) the water system causing the cases was free of ST2858 at the time of sampling, likely due to disinfection, or (iii) ST2858 represented a minor proportion of the *L. pneumophila* community within the source system, reducing the likelihood of its detection with plate culture and/or being chosen for further characterization. The probability of the latter was initially deemed likely when a co-infection with ST2858 and ST378 was reported, and subsequent environmental sampling found ST378 in the HWDS of HF-A. Genomic analysis of the ST378 isolates from HF-A and ST378 from other healthcare facilities supports the hypothesis that the source of ST378 from the case with the co-infection was the HWDS of HF-A. If the patient with the co-infection was infected by both STs at the same time, then ST2858 likely co-occurred with ST378 in the HWDS of HF-A. Further screening of 187 isolates and 11 DNA extracts from HF-A with our PCR test was unsuccessful. This indicates that less than 0.5% of isolates from this HWDS are ST2858 and that the population of ST2858 may only reach a detectable and transmissible level on very rare occasions, which might not have been captured by our sampling efforts.

It is also important to consider that epidemiological investigation indicated that some cases had a most likely exposure setting outside HF-A (Table 1), suggesting that the source of ST2858 releases aerosols that could be inhaled outside HF-A, advocating against the HWDS being the source of ST2858. The sources of nosocomial cases are most often the water distribution system of the hospital, but WCT has been identified as the source in a few cases (41, 42). WCTs of HF-A were sampled, but other STs were detected in them, such as ST-1 (Table S1). Screening of 412 isolates and 41 DNA samples from other sources was also unsuccessful in identifying a source of ST2858. This indicates that ST2858 might be an extremely rare strain in Quebec. Nevertheless, HF-A WCTs cannot be ruled out as the source, because it is expected that the ST2858 would represent a minor fraction of the *Lp* population and because of timing issues with sampling, as discussed above for the HWDS of HF-A. The patient with the co-infection could have been infected by the two strains at different times and from different sources. Co-infections by different species of *Legionella*, by different serogroups of *L. pneumophila*, and by different STs were previously reported (43). Sputum analysis of the LD patients by 16S rRNA sequencing detected co-infection by *Lp* and other species (44). The prevalence of co-infections in LD is likely underappreciated because only one isolate from clinical samples is typically typed, and more efforts are needed to uncover the dynamics of co-infection in LD. The exposure setting of the patient with the co-infection was clearly HF-A and classified as a nosocomial case, indicating that the source of ST2858 was most likely a source nearby HF-A, such as its WCTs. This could indicate co-infection from different sources, ST378 from the HWDS and ST2858 from the WCTs of HF-A. This is supported by the fact that no more cases caused by ST2858 were identified after the management of the HWDS and WCTs of HF-A were improved, including through *in situ* monochloramination of the HWDS starting in 2022 (45). Alternatively, ST2858 could reside in a hidden niche connected to both the HWDS and WCTs of HF-A. A nosocomial outbreak in 1985 was caused by the hospital WCT, and isolates of the same subgroup as the clinical isolates were also found in the WCT make-up water (41). Colonization of a small area of the main drinking water pipe, such as a dead-end, supplying both the HWDS and the WCTs of HF-A could enable ST2858 to be sporadically aerosolized by both systems, explaining the nosocomial and community-acquired cases and the difficulty in finding it in these systems, as it would not be a resident of them.

Phylogenetic and pangenome analysis of both STs showed high genetic diversity between them, suggesting different genomic backgrounds and preferred environmental niches. Contrary to ST2858, comparative analysis of ST378 genomes showed diverse genomes. ST378 seems endemic in Montréal and was recovered from the HWDS of four different Montreal healthcare facilities. Overall, phylogenetic analysis revealed that the ST378 isolates cluster according to their site of isolation. Pairwise comparison revealed that the number of SNPs is not always different between strains from the same facility and those from different facilities. For example, SPF600 from HF-A shows 60 SNPs compared with SPF599 from the same facility, but also 61 SNPs compared with SPF639 from HF-C. This means that the source of the clinical ST378 would not be possible to identify based on genomic comparison with only one environmental isolate from each facility and could complicate source attribution for a patient transferred from one facility to another during their incubation period. Similar diversity among isolates from large HWDS has been reported by other groups (46). Therefore, source investigation benefits from including several isolates from the same source to determine diversity within the residing *Lp* community.

Overall, the accessory genome of ST378 was small, with 2,699 core genes and 519 accessory genes. Of those, 262 are cloud genes because they are present in less than 15% of genomes. Most of the latter are present on MGE acquired by specific strains: 73 in SPF635 and SPF636, 60 in SPF638 and 98 in SPF581. In SPF581, the putative plasmid identified in SPF581, the isolate responsible for mixed infection harbored genes encoding conjugative elements. This observation and the fact that other contemporary strains (such as SPF600) do not possess it suggest it was most likely acquired through a recent HGT event. HGT plays an important role in the diversity of *L. pneumophila* and may enable it to rapidly adapt and persist in specific environments through acquisition of new genes (47, 48). Furthermore analysis identified the presence of an extra copy of *copA* as well as the *mmcO* gene on the plasmid. Similarly, an extra copy of these genes was also detected in another environmental isolate SPF638. However, in that case, the genetic region was found integrated in the chromosome. In *L. pneumophila*, *copA*-encoded copper-translocating P_IB_-type ATPase has been shown to mediate copper resistance under high copper conditions (49). It is located within a 100-kilobase mobile genetic element, the excision of which is controlled by the regulatory activity of the small RNA binding protein, Hfq (49). However, in its episomal form, it is linked with higher expression of *copA*, hence an increased tolerance to copper, due to gene dosage. It was previously speculated that microevolution could be observed when *L. pneumophila* strains are subjected to different selection pressures (35). It is likely that the harsh conditions in the HWDS of HF-A, including high temperature (>55°C) and average copper concentration of 362 µg/L selected for the acquisition of copper resistance genes and of the plasmid, increasing its ability to persist in this system. Indeed, SPF581 shows increased resistance to copper *in vitro* compared to other ST378 isolates collected at the same time from the same water system. This is likely due to the increased expression of *copA* and *mmcO* from the multicopy plasmid it carries. This finding and previous investigations suggesting high genomic plasticity and variation in *L. pneumophila* and other *Legionella* species (50, 51) due to the acquisition of new genes introduced by HGT and recombination confirm the importance of this process in *L. pneumophila* evolution and adaptation.

Since ST2858 is rare in the environment compared to ST378, how can we explain that there are more cases caused by ST2858 than ST378? ST2858 enrichment in clinical samples may indicate higher clinical virulence, which is supported by the presence of *lag-1* (52) in its genome, but not in ST378. This gene is enriched in clinical isolates of *Lp* and confers complement resistance by acetylation of LPS (52). Therefore, while ST378 seems more adapted to persistence in HWDS, ST2858’s potential higher virulence might make it more likely to cause severe infections that are diagnosed. Alternatively, ST2858 might be more easily aerosolized than ST378 (53); however, no gene associated with this trait is currently known. Nevertheless, genetic diversity in *Lp* does affect the frequency of detection in both environmental and clinical samples.

### Conclusion

We applied WGS to shed light on a recurrent outbreak associated with ST2858 and centered on HF-A. Although the source could not be identified directly by confirming the presence of live ST2858 isolates in a water system, the epidemiological investigation and the comparative genetic analysis together strongly suggest that a single common environmental source of ST2858 caused the 2019 and 2021 outbreaks. The presence of both nosocomial and community-acquired cases could be explained by ST2858 colonizing a WCT of HF-A and ST378 the HWDS, with the co-infection case being infected by separate events. Our study urges for additional research on the prevalence of co-infection in LD and diversity of *Lp* within patients. The absence of nosocomial cases after the 2021 outbreak suggests that the source is now controlled. We also described a novel approach, based on targeting unique genes by PCR, to screen a large number of samples for a specific strain. Application of this method requires that historical strains are isolated and stored from the mandatory compliance testing. Diversity within the ST378 population was substantially higher than ST2858, potentially reflecting the successful colonization of large water systems by ST378, and hence higher environmental pressure and higher substitution rate in their genome. Finally, we showed that this genomic diversity could lead to phenotypic diversity, exemplified by higher copper resistance mediated by the acquisition of a plasmid. Overall, this study supports the use of WGS for LD outbreak investigations.

## MATERIALS AND METHODS

### Culture and serogroup testing

Clinical and environmental isolates (Table 1; Table S1) were grown on CYE (ACES-buffered charcoal yeast extract) agar plates (Sigma-Aldrich) and incubated at 37°C for 3 days. Liquid cultures were grown in AYE broth (CYE without charcoal or agar) at 37°C with shaking. All isolates were serogrouped using the Antiserum *Legionella pneumophila* group 1 kit (Deika Seiken).

### WGS

For WGS, genomic DNA was extracted from broth culture using the Wizard Genomic DNA purification kit (Promega) following the manufacturer’s instructions. The DNA quality and quantity of extracts were measured using the Nanodrop spectrophotometer (Thermo Scientific NanoDrop 1000), and DNA integrity was assessed via gel electrophoresis. Short-read and long-read sequencing was performed using Illumina MiSeq and Oxford Nanopore Technologies (ONT) platforms, respectively. For Illumina sequencing, DNA was sequenced using MiSeq PE250 or NovaSeq 6000 using PE150 chemistry. Libraries were prepared by the McGill Genome Center. For ONT sequencing, libraries were prepared by the Nanopore Sequencing Platform at the CHU-Sainte-Justine Research Center. High molecular weight DNA was prepared using the ONT SQK-RBK004 or SQK-RBK110.96 rapid barcoding protocols that were modified to use 0.8 µL of rapid adapter (RAP) per sample. Samples were sequenced on R9.4.1 MinION or PromethION flowcells (see Table S2).

### Quality control, genome assembly, and annotation

Illumina paired-end reads were quality-checked using FastQC (54). Adapters and low-quality reads (Phred < 25) were filtered out using fastp with default parameters (55). For ONT sequencing, reads were base called with either Guppy v6.2.1 (MinION) or v6.4.6 (PromethION) in super-high accuracy mode with configuration file “dna_r9.4.1_450bps_sup.cfg” and default demultiplexing parameters were applied. Basic quality metrics and statistics of the long reads were checked using Nanoplot before downstream analyses (56).

Short-read assemblies were produced with SPAdes (57). Hybrid assemblies were generated by combining short and long reads, with Illumina short reads scaffolded on ONT long reads using NanoForms (58). The quality of the assemblies was assessed using Bandage (59) and MiGA (60) to evaluate percent completeness, and percent contamination, and confirm taxonomic assignment. Details regarding the type of assembly and the quality control parameters for each isolate are shown in Table S2. *In silico* typing of isolates was performed with legsta (61). Prokka was used for genome annotation using Philadelphia-1 as reference (62). Hybrid assemblies were compared using Mauve (63) to identify conserved genomic regions and rearrangements. For all analysis tools, default parameters were used unless otherwise specified. Assembled genomes and raw reads are available from GenBank under project number PRJNA1186735.

### Phylogenetic analysis and recombination detection

For phylogenetic analysis of both ST2858 and ST378 isolates, kSNP4 was used (v4.1), which derives core genome SNPs based on k-mer analysis of sequences (64). kSNP4 was run on assembled genomes using kmer size of 17 as determined by Kchooser. Parsimony trees were visualized and annotated with FigTree (v1.4.4) (65). Gubbins (66) was used for detecting recombination and phylogenetic analysis. Briefly, assembled genome sequences of the isolates were aligned to the complete reference genomes of the respective STs (SPF590 for ST2858 and SPF637 for ST378) using SKA2 (67). The alignment was then subjected to Gubbins analysis for recombination detection and filtering. The analysis was performed using the parameters --tree-builder: iqtree, --bootstrap: 1000, and --transfer-bootstrap: TRUE, with all other parameters at default. This bootstrapping approach has been shown to yield higher support values for the inferred branches (68). Pairwise SNP difference between all genomes was calculated using pairsnp (https://github.com/gtonkinhill/pairsnp).

### Pangenome analysis

Pangenome analyses were conducted on the annotated genomes using Panaroo (69). The analysis was run using sensitive mode to keep plasmids and other mobile genetic elements under consideration. Furthermore, the gene_presence_absence.csv file generated by Panaroo was used to identify the core and accessory genes in the genomes.

### Detection of AMR genes

All genomes were screened for antimicrobial resistance. AMR genes were identified using ABricate (70) against the CARD (71).

### Screening historical isolates and DNA extracts for ST2858

To increase the screening capacity of putative water sources, a simple PCR assay was developed to amplify a unique gene of ST2858. Assembled and Prokka-annotated genomes of eight isolates of ST2858, four of ST1, four of ST378, and five other STs (Table S1) were analyzed with Roary (72) to determine which genes were unique to ST2858. Genes with uncharacterized functions, which are less likely essential and so unique to ST2858, were compared against the NCBI BLAST nucleotide database. Those with few hits against other *Legionella* non-*pneumophila* or other bacterial species were chosen as prime PCR target candidates. Primers were designed with Benchling, aiming for product size of 200–500 bp and high specificity as determined with NCBI primer blast. Six primer pair candidates were ordered for testing (Table S8).

Primers were tested with cell lysates of target and non-target bacteria (Table S1) using OneTaq DNA Polymerase (New England Biolabs) in 50 µL reactions according to the manufacturer’s instructions with 2 µL of template. DNA template was obtained by mixing one colony in 25 µL of 0.1 N NaOH and incubating at room temperature for 20 min. Then, 25 µL of 1 M Tris pH 7.4 and 450 µL of sterile Milli-Q were added. DNA extracted from ST2858 isolates and 2 µL of sterile PCR-grade water were used as a positive and negative control respectively in all PCR amplifications. Tubes were placed in pre-heated Veriti 96-well thermal cycler (Applied Biosystems) and subjected to the following conditions: initial denaturation at 95°C for 30 s, 30 cycles of denaturation at 95°C for 15 s, annealing at 58°C or 62°C depending on primer for 15 s, elongation at 68°C for 40 s and a final elongation at 68°C for 5 min. Products were analyzed by gel electrophoresis in 1.2% agarose gel with ethidium bromide (0.6 µg/mL) and visualized under UV light in E-Box gel doc (Vilber).

Isolates of serogroup 1 that were collected by private laboratories between September 2019 and December 2021 during compliance testing were chosen for screening, resulting in 412 isolates from 12 administrative regions in the province of Quebec (Table S9). In addition, 187 isolates collected by our team using Legiolert and plate culture (73) (74) were tested. DNA was extracted from the isolates as described above. Finally, 52 DNA extracts from water samples collected in 2021 and 2022 were also tested. DNA was extracted from 1 L of water filtered through a 0.22 µm pore-size mixed cellulose-ester membrane filter (Millipore). The filters were frozen at −20°C until processed for DNA extractions with the MP FastDNA Spin Kit DNA extraction kit per the manufacturer’s instructions. As a control, the presence of the gene *mip* was confirmed by PCR amplification according to ESGLI protocol (75).

### Copper resistance

Isolates were grown in 1 mL AYE cultures overnight at 37°C. After incubation, isolates were pelleted at 5,000 × *g* for 5 min, then washed and resuspended in Fraquil, a defined low-nutrient medium that simulates freshwater (76), to a final concentration of 10^8^ cells/mL and incubated at room temperature overnight. In a 24-well plate, 990 µL of each isolate was added to the wells in triplicate, and then 10 µL of 8 mM CuCl_2_ was added to each well, for a final concentration of 0.08 mM CuCl_2_. As a control, 10 µL of Fraquil was added instead of copper to a different triplicate of the isolate in each well in another 24-well plate. The plates were incubated at room temperature for 4 h. A CFU count of both the control- and copper-exposed conditions was taken to determine survival.

## SUPPLEMENTAL MATERIAL

Supplemental figures spectrum.00513-25-s0001.pdf Fig. S1 to S6.

Supplementary Tables S1, S2, S4, S5, S7, S8, S9 spectrum.00513-25-s0002.pdf Tables S1, S2, S4, S5, and S7 to S9.

Table S3 spectrum.00513-25-s0003.xlsx Genome annotation of strains included in this study.

Table S6 spectrum.00513-25-s0004.csv Details of the presence and absence of annotated orthologous genes across ST378 isolates analyzed using Panaroo.

ASM does not own the copyrights to Supplemental Material that may be linked to, or accessed through, an article. The authors have granted ASM a non-exclusive, world-wide license to publish the Supplemental Material files. Please contact the corresponding author directly for reuse.
