# Supplementary material for: Comparative genome analysis investigation of nosocomial and community-acquired cases of Legionnaires’ disease caused by ST2858 and ST378
Source: Microbiol Spectr. 2025 Jun 9;13(7):e00513-25. doi: 10.1128/spectrum.00513-25 (PMC12211059; doi:10.1128/spectrum.00513-25)
Supplement: Supplemental figures — Fig. S1 to S6. [file spectrum.00513-25-s0001.pdf]

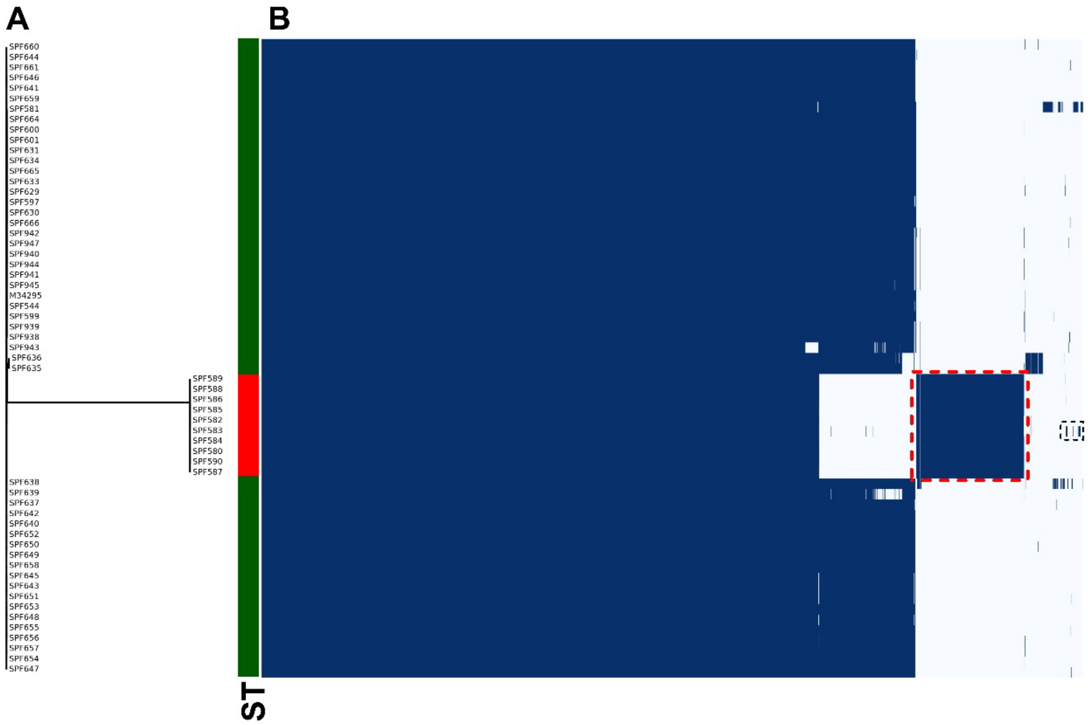

Figure S1: Pangenome analysis of ST2858 and ST378 isolates. (A) Phylogenetic tree constructed based on core genes. (B) Pangenome matrix illustrating gene presence (blue) and absence (white). Genes unique to all ST2858 isolates are highlighted with a red rectangle (n = 485), while genes found exclusively in isolate one ST2858 isolate (SPF583) are highlighted with a black rectangle (n = 22).

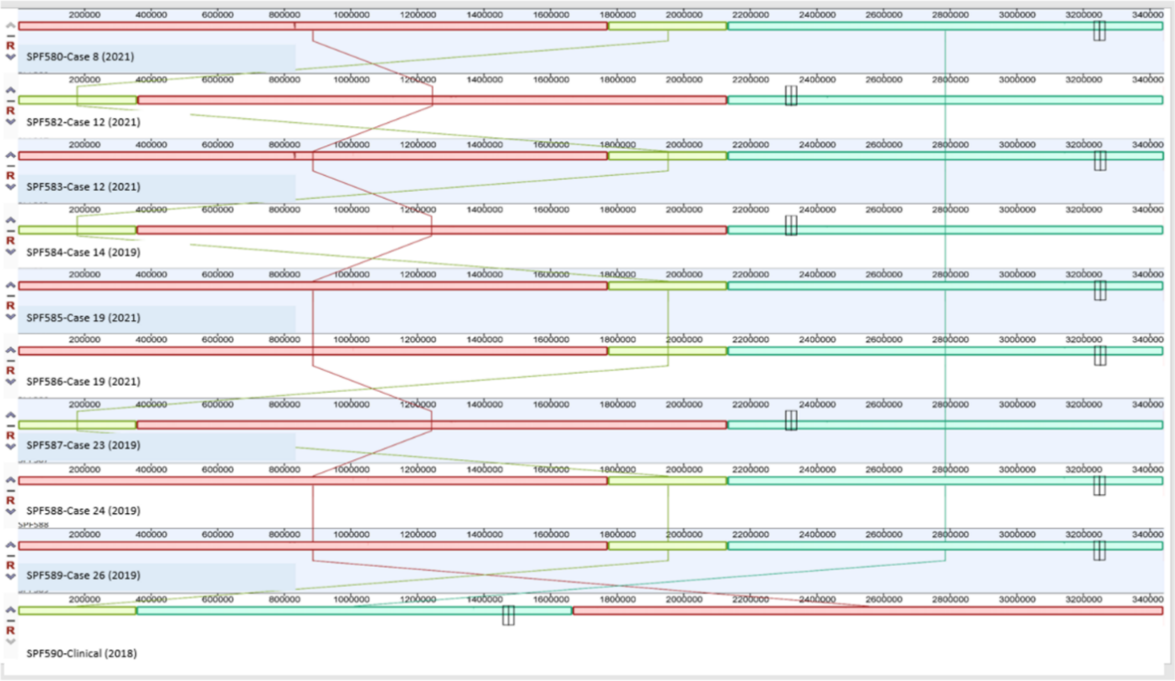

Figure S2: Multiple whole-genome alignment of *L. pneumophila* ST2858 complete genomes generated by progressive Mauve aligner and visualized using Mauve viewer. Each genome's panel is annotated with source and year of isolation. Lines joining each similarly colored block showed homology in the sequence among genomes. shows that a large portion of each ST2858 genome was homologous and identified as Local Colinear Blocks (LCBs).

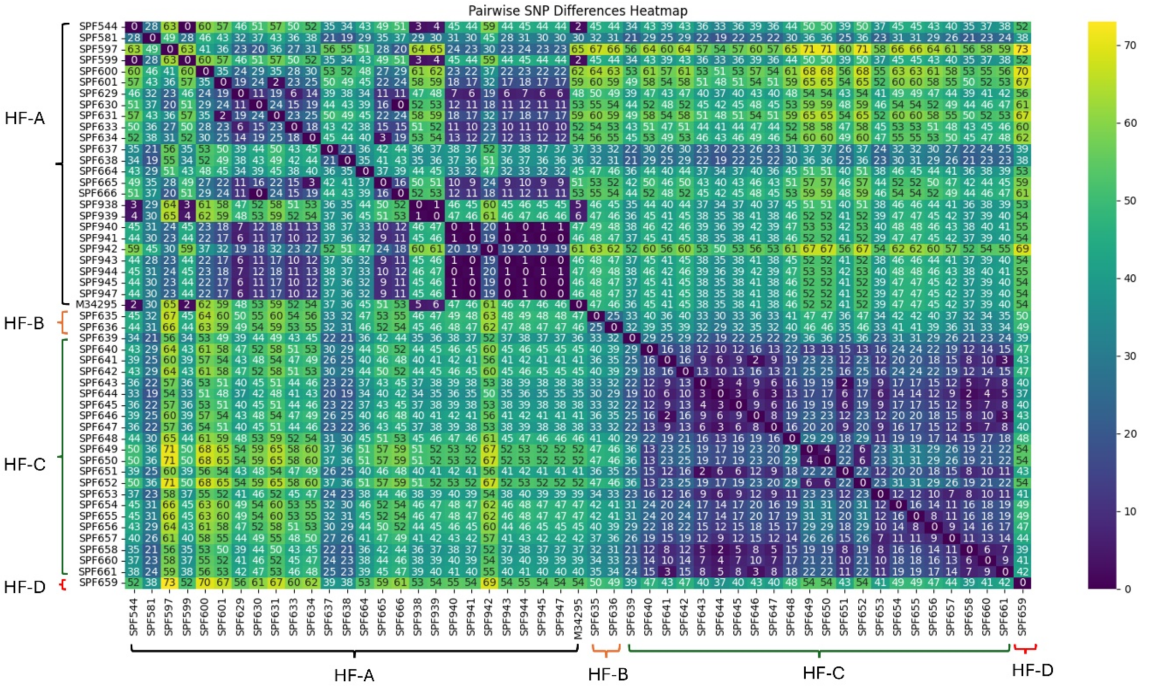

Figure S3: Heatmap showing pairwise SNP distance between ST378 genomes outside the recombination regions. Isolates are marked according to the source (HF).

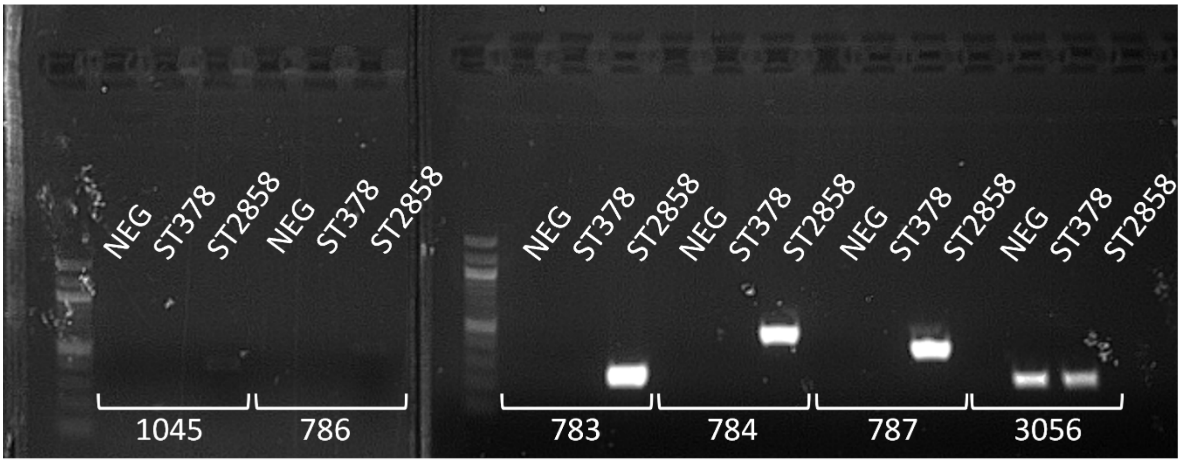

Figure S4: Initial testing of candidate primers for ST2858 screening. Cell lysates were made from ST378 (SPF544) and ST2858 (SPF584). Nuclease free water served as a negative control (NEG). Products were run on 1.2% agarose gel with ethidium bromide against NEB 1 kg ladder. Primers 783, 784 and 787 only amplified genes from ST2858 and were chosen for further testing. A 100 bp ladder (NEB) was used: the top bright band is 1,000 bp, the bottom bright band is 500 pb.

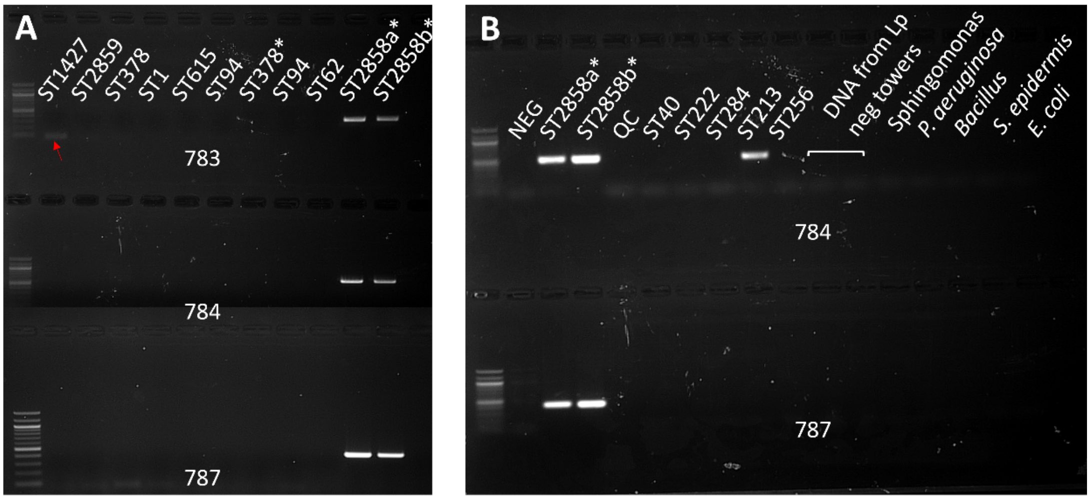

Figure S5: Testing ST2858 specific primers against A) cell lysates of other sequence types of *L. pneumophila* and B) cell lysates of other sequence types of L. pneumophila and bacteria commonly found in cooling towers (cell lysates or DNA extract from *L. pneumophila* negative towers. Samples were run against NEB 1kb ladder and * indicates clinical strains. Primers 783 had some amplification with ST1427 (red arrow) and so was not included in the next round of testing. Primer 784 reacted with ST213 of *L. pneumophila*. Primer 787 had no cross-reactivity and so was chosen as the final primer for ST2858 screening. A 100 bp ladder (NEB) was used: the top bright band is 1,000 bp, the bottom bright band is 500 pb.

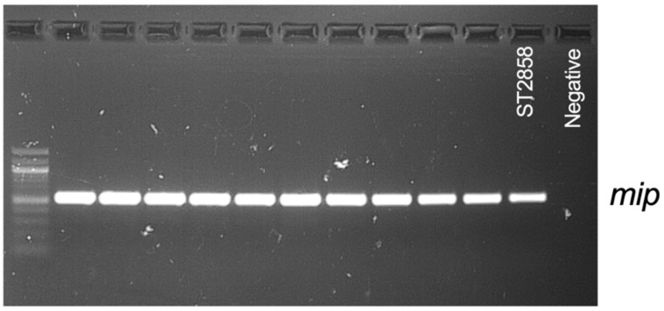

Figure S6: PCR on *mip* gene was performed to confirm the successful DNA extraction from cell lysates used for ST2858 screening. A 100 bp ladder (NEB) was used: the top bright band is 1,000 bp, the bottom bright band is 500 pb.
