# Supplementary material for: Comparative genome analysis investigation of nosocomial and community-acquired cases of Legionnaires’ disease caused by ST2858 and ST378
Source: Microbiol Spectr. 2025 Jun 9;13(7):e00513-25. doi: 10.1128/spectrum.00513-25 (PMC12211059; doi:10.1128/spectrum.00513-25)
Supplement: Supplementary Tables S1, S2, S4, S5, S7, S8, S9 — Tables S1, S2, S4, S5, and S7 to S9. [file spectrum.00513-25-s0002.pdf]

Supplementary materials

Table S1: Summary of the other isolates/bacteria used in the study.

Strain ID	Type	Isolation site[c]	Year of isolation	ST	Name	Source
SPF325	Clinical	NA	2006	ST62	NA	Lévesque et al. 2014
SPF543[ab]	Environmental	HWDS (D)	2021	ST1427	NA	Matthews et al. 2022
SPF545[ab]	Environmental	CT (E)	2021	ST1	NA	This study
SPF546[ab]	Environmental	HWDS (D)	2021	ST1427	NA	Matthews et al. 2022
SPF578[ab]	Clinical	NA	2021	ST615	NA	This study
SPF579[ab]	Clinical	NA	2019	ST94	NA	This study
SPF592[a]	Environmental	CT (F)	2021	ST1	NA	This study
SPF593[a]	Environmental	CT (HF-A)	2021	ST1	NA	This study
SPF594[a]	Environmental	CT (G)	2021	ST1	NA	This study
SPF373[b]	NA	NA	NA	NA	*Staphylococcus epidermidis*	Lab strain bank
SPF427[b]	NA	NA	NA	NA	*Bacillus*	Lab strain bank
SPF445[b]	NA	NA	NA	NA	*Pseudomonas aeruginosa*	Lab strain bank
SPF447[b]	NA	NA	NA	NA	*Sphingomonas*	Lab strain bank
DH5a[b]	NA	NA	NA	NA	*Escherichia coli*	Lab strain bank

a) used in primer design for ST2858 environmental screening

b) used in primer testing for ST2858 environmental screening

c) HWDS, hot water distribution system; CT, cooling towers; HF-A, healthcare facility A.

NA: not applicable for this study.

Table S2: Summary of general assembly characteristics of the isolates

				Bandage				MiGA		
**Strain ID**	**ST**	**Sequencing technology**	**Assembly type**	N50 (bp)	Depth	estimated length (bp)	dead ends (%)	Contamination (%)	Completeness (%)	Accession number
SPF544	ST378	Illumina MiSeq, Oxford Nanopore MinION	Hybrid	300392	1.00x	3349892	0.00%	1.9	100	CP174245
SPF545	ST1	Illumina MiSeq	Short-read	160,262	18.7x	3,658,955	0.10%	1.9	100	JBJUJL000000000
SPF578	ST615	Illumina MiSeq	Short-read	161,612	20x	3,581,441	0.40%	0.9	100	JBJUJK000000000
SPF579	ST94	Illumina MiSeq	Short-read	213,353	16.5x	3,557,620	1.09%	0.9	100	JBJUJJ000000000
SPF580	ST2858	Illumina MiSeq, Oxford Nanopore MinION	Hybrid	3442463	1.00x	3442463	0.00%	1.9	100	CP174244
SPF581	ST378	Illumina MiSeq, Oxford Nanopore MinION	Hybrid	3363915	1.00x	3630243	0.00%	1.9	100	CP176367-CP176368
SPF582	ST2858	Illumina MiSeq, Oxford Nanopore MinION	Hybrid	3442874	1.00x	3442874	0.00%	1.9	100	CP174243
SPF583	ST2858	Illumina MiSeq, Oxford Nanopore MinION	Hybrid	3442878	1.00x	3442878	0.00%	0.9	100	CP174242
SPF584	ST2858	Illumina MiSeq, Oxford Nanopore MinION	Hybrid	3441795	1.00x	3441795	0.00%	1.9	100	CP174241
SPF585	ST2858	Illumina MiSeq, Oxford Nanopore MinION	Hybrid	3441795	1.00x	3441795	0.00%	0.9	100	CP174240
SPF586	ST2858	Illumina MiSeq, Oxford	Hybrid	3442832	1.00x	3442832	0.00%	0.9	100	CP174239

		Nanopore MinION								
SPF587	ST2858	Illumina MiSeq, Oxford Nanopore MinION	Hybrid	3442832	1.00x	3442832	0.00%	0.9	100	CP174238
SPF588	ST2858	Illumina MiSeq, Oxford Nanopore MinION	Hybrid	3441777	1.00x	3441777	0.00%	0.9	100	CP174237
SPF589	ST2858	Illumina MiSeq, Oxford Nanopore MinION	Hybrid	3441767	1.00x	3441767	0.00%	0.9	100	CP174236
SPF590	ST2858	Illumina MiSeq, Oxford Nanopore MinION	Hybrid	3441991	1.00x	3441891	0.00%	0.9	100	CP174235
SPF592	ST1	Illumina MiSeq	Short-read	138,207	22.7x	3,496,663	1.16%	0.9	100	JBJUJI000000000
SPF593	ST1	Illumina MiSeq	Short-read	160,264	27.0x	3,615,138	0.00%	0.9	100	JBJUJH000000000
SPF594	ST1	Illumina MiSeq	Short-read	128,995	19.7x	3,840,197	0.59%	0.9	100	JBJUJG000000000
SPF597	ST378	Illumina MiSeq, Oxford Nanopore PromethION	Hybrid	303411	1.00x	3332670	0.00%	0.9	100	CP174446
SPF599	ST378	Illumina MiSeq	Hybrid	300392	1.00x	3350283	0.00%	0.9	100	JBJUJF000000000
SPF600	ST378	Illumina MiSeq, Oxford Nanopore PromethION	Hybrid	303411	1.00x	3330665	0.00%	0.9	100	CP174234
SPF601	ST378	Illumina MiSeq, Oxford Nanopore PromethION	Hybrid	303411	1.00x	3328382	0.00%	0.9	100	CP174233
SPF629	ST378	Illumina MiSeq	Short-read	303412	21.7x	3334684	2.78	0.9	100	JBJUJE000000000
SPF630	ST378	Illumina MiSeq, Oxford	Hybrid	300391	1.00x	3332805	0.00%	0.9	100	CP174232

		Nanopore PromethION								
SPF631	ST378	Illumina MiSeq, Oxford Nanopore PromethION	Hybrid	303,411	1.00x	3333159	0.00%	0.9	100	CP174445
SPF633	ST378	Illumina MiSeq, Oxford Nanopore PromethION	Hybrid	303412	1.00x	3334419	0.00%	0.9	100	CP174231
SPF634	ST378	Illumina MiSeq, Oxford Nanopore PromethION	Hybrid	300392	1.00x	3335285	0.00%	0.9	100	CP174230
SPF635	ST378	Illumina NovaSeq 6000	Short-read	248576	51.8x	3304811	3.75%	0.9	100	JBJUJD000000000
SPF636	ST378	Illumina NovaSeq 6000	Short-read	248577	56.4	3384889	3.20%	0.9	100	JBJUJC000000000
SPF637	ST378	Illumina MiSeq, Oxford Nanopore PromethION	Hybrid	303411	1.00x	3335605	0.00%	0.9	100	CP174229
SPF638	ST378	Illumina MiSeq, Oxford Nanopore PromethION	Hybrid	134615	1.00x	3424006	0.00%	0.9	100	CP174228
SPF639	ST378	Illumina MiSeq, Oxford Nanopore PromethION	Hybrid	300392	1.00x	3231105	0.00%	0.9	100	CP174227
SPF640	ST378	Illumina MiSeq, Oxford Nanopore PromethION	Hybrid	248358	1.00x	3332880	0.00%	0.9	100	CP174226
SPF641	ST378	Illumina MiSeq, Oxford Nanopore PromethION	Hybrid	300395	1.00x	3331280	0.00%	0.9	100	CP174225
SPF642	ST378	Illumina MiSeq, Oxford Nanopore PromethION	Hybrid	303410	1.00x	3333407	0.00%	0.9	100	CP174224

SPF643	ST378	Illumina MiSeq, Oxford Nanopore PromethION	Hybrid	303411	1.00x	3320508	0.00%	0.9	100	CP174444
SPF644	ST378	Illumina MiSeq, Oxford Nanopore PromethION	Hybrid	219576	1.00x	3332383	3.18%	0.9	100	CP174223
SPF645	ST378	Illumina MiSeq, Oxford Nanopore PromethION	Hybrid	248357	1.00x	3322746	0.00%	0.9	100	CP174222
SPF646	ST378	Illumina MiSeq, Oxford Nanopore PromethION	Hybrid	300395	1.00x	3331291	0.00%	0.9	100	CP174221
SPF647	ST378	Illumina MiSeq, Oxford Nanopore PromethION	Hybrid	303411	1.00x	3332326	0.00%	0.9	100	CP174220
SPF648	ST378	Illumina MiSeq	Short-read	303414	20.2x	3322564	0.65%	0.9	100	JBJUJB000000000
SPF649	ST378	Illumina MiSeq, Oxford Nanopore PromethION	Hybrid	303410	1.00x	3332581	0.00%	0.9	100	CP174219
SPF650	ST378	Illumina MiSeq, Oxford Nanopore PromethION	Hybrid	300395	1.00x	3331166	0.00%	0.9	100	CP174218
SPF651	ST378	Illumina MiSeq, Oxford Nanopore PromethION	Hybrid	270911	1.00x	3322314	0.00%	0.9	100	CP174217
SPF652	ST378	Illumina MiSeq, Oxford Nanopore PromethION	Hybrid	244922	1.00x	3330400	0.00%	0.9	100	CP174216
SPF653	ST378	Illumina MiSeq, Oxford Nanopore PromethION	Hybrid	303414	1.00x	3331758	0.00%	0.9	100	CP174215

SPF654	ST378	Illumina MiSeq, Oxford Nanopore PromethION	Hybrid	197814	1.00x	3330097	0.00%	0.9	100	CP174214
SPF655	ST378	Illumina MiSeq	Short-read	300392	13.7x	3331879	0.00%	0.9	100	JBJUJA000000000
SPF656	ST378	Illumina MiSeq	Short-read	248358	14.0x	3331786	1.49%	0.9	100	JBJUIZ000000000
SPF657	ST378	Illumina MiSeq, Oxford Nanopore PromethION	Hybrid	303413	1.00x	3333528	0.00%	0.9	100	CP174213
SPF658	ST378	Illumina MiSeq	Hybrid	300395	1.00x	3331108	0.00%	0.9	100	CP174212
SPF659	ST378	Illumina MiSeq	Short-read	303411	26.5x	3334156	1.57%	0.9	100	JBJUIY000000000
SPF660	ST378	Illumina MiSeq, Oxford Nanopore PromethION	Hybrid	303411	1.00x	3332144	0.00%	0.9	100	CP174443
SPF661	ST378	Illumina MiSeq, Oxford Nanopore PromethION	Hybrid	196639	1.00x	3330163	2.55%	0.9	100	CP174442
SPF664	ST378	Illumina MiSeq, Oxford Nanopore PromethION	Hybrid	247117	1.00x	3337178	0.00%	0.9	100	CP174211
SPF665	ST378	Illumina MiSeq, Oxford Nanopore PromethION	Hybrid	303411	1.00x	3334675	0.00%	0.9	100	CP174210
SPF666	ST378	Illumina MiSeq, Oxford Nanopore PromethION	Hybrid	300391	1.00x	3331926	0.00%	0.9	100	CP174209
SPF938	ST378	Illumina NovaSeq 6000	Short-read	197255	63.1x	3355495	2.48%	0.9	100	JBJUIX000000000
SPF939	ST378	Illumina NovaSeq 6000	Short-read	197255	62.8x	3361459	0.64%	0.9	100	JBJUIW000000000
SPF940	ST378	Illumina NovaSeq 6000	Short-read	248578	64.8x	3347905	5.76%	0.9	100	JBJUIV000000000

SPF941	ST378	Illumina NovaSeq 6000	Short-read	248578	77.3x	3348666	1.37%	0.9	100	JBJUIU000000000
SPF942	ST378	Illumina NovaSeq 6000	Short-read	248578	53.3x	3343295	0.91%	0.9	100	JBJUIT000000000
SPF943	ST378	Illumina NovaSeq 6000	Short-read	303294	116.1x	3267446	14.47%	0.9	100	JBJUIS000000000
SPF944	ST378	Illumina NovaSeq 6000	Short-read	248578	47.1x	3347056	0.75%	0.9	100	JBJUIR000000000
SPF945	ST378	Illumina NovaSeq 6000	Short-read	248578	75.3x	3349073	8.86%	0.9	100	JBJUIQ000000000
SPF947	ST378	Illumina NovaSeq 6000	Short-read	248578	64.0x	3350469	7.89%	0.9	100	JBJUIP000000000
M34295	ST378	Illumina MiSeq	Short-read	300392	44.8x	3349074	2.14%	0.9	100	JBJUIO000000000

Table S4: Summary of unique genes and their functional annotation in ST2858

Gene name	Annotation
group_2085	hypothetical protein
lvrB_1	LvrB
group_1737	hypothetical protein
group_1686	hypothetical protein
group_1675	hypothetical protein
group_1674	hypothetical protein
group_1673	hypothetical protein
group_1672	hypothetical protein
group_1671	hypothetical protein
group_1670	transposase
group_1669	transposase
group_1668	transposase
group_1667	transposase
group_1666	transposase
group_1665	transposase IS4 family Tn5
group_1664	transposase IS4 family Tn5
group_1663	transposase IS4 family Tn5
group_1662	transposase IS4 family Tn5
group_1661	transposase IS4 family Tn5
group_1660	transposase (IS652)
group_1659	transposase (IS652)
group_1658	transposase IS4 family Tn5
group_1657	IS630 family transposase ISSpu23
group_1656	IS630 family transposase ISSpu23
group_1655	IS630 family transposase ISSpu23
group_1654	IS630 family transposase ISSpu23

group_1652	IS630 family transposase ISSpu23
group_1651	IS630 family transposase ISSpu23
group_1650	IS630 family transposase ISSpu23
group_1649	IS630 family transposase ISSpu23
group_1648	IS630 family transposase ISSpu23
group_1646	transposase IS911
group_1645	transposase IS911
group_1644	transposase IS911
group_1643	transposase IS911
group_1642	transposase IS911
group_1641	transposase IS911
group_1639	transposase IS911
group_1638	transposase IS911
group_1637	IS3 family transposase ISLpn7
group_1636	IS3 family transposase ISLpn7
group_1635	IS3 family transposase ISLpn7
group_1634	IS3 family transposase ISLpn7
group_1633	IS3 family transposase ISLpn7
group_1632	IS3 family transposase ISLpn7
group_1630	IS3 family transposase ISLpn7
group_1629	IS3 family transposase ISLpn7
group_1624	hypothetical protein
group_1623	hypothetical protein
group_1621	hypothetical protein
group_1615	hypothetical protein
group_1614	hypothetical protein
group_1611	hypothetical protein
group_1610	hypothetical protein

group_1609	hypothetical protein
group_1604	hypothetical protein
group_1603	hypothetical protein
group_1602	hypothetical protein
group_1600	hypothetical protein
group_1599	hypothetical protein
group_1598	oxidase
group_1596	hypothetical protein
group_1591	hypothetical protein
group_1590	transposase ORF-A IS-type
group_1586	transposase (ISmav2)
group_1584	hypothetical protein
group_1576	hypothetical protein
group_1575	hypothetical protein
group_1574	hypothetical protein
group_1569	hypothetical protein
group_1568	hypothetical protein
group_1567	hypothetical protein
group_1566	hypothetical protein
group_1563	hypothetical protein
group_1562	hypothetical protein
group_1560	hypothetical protein
group_1556	hypothetical protein
group_1549	hypothetical protein
group_1548	hypothetical protein
group_1544	hypothetical protein
group_1538	hypothetical protein
group_1537	hypothetical protein

group_1532	hypothetical protein
group_1526	hypothetical protein
group_1525	hypothetical protein
group_1519	hypothetical protein
group_1515	prophage regulatory protein-like protein
group_1514	hypothetical protein
group_1513	hypothetical protein
group_1512	hypothetical protein
group_1501	hypothetical protein
group_1496	cold shock domain family protein
group_1495	hypothetical protein
group_1489	hypothetical protein
group_1486	hypothetical protein
group_1479	hypothetical protein
group_1477	hypothetical protein
group_1476	hypothetical protein
group_1475	hypothetical protein
csrA_3	Translational regulator CsrA
group_1466	hypothetical protein
group_1461	hypothetical protein
group_1457	transposase
group_1456	hypothetical protein
group_1454	hypothetical protein
group_1453	hypothetical protein
group_1448	hypothetical protein
group_1445	hypothetical protein
group_1441	hypothetical protein
group_1440	hypothetical protein

group_1439	hypothetical protein
group_1438	hypothetical protein
group_1437	transposase TnpA
group_1436	hypothetical protein
group_1435	hypothetical protein
group_1433	hypothetical protein
group_1431	hypothetical protein
group_1430	hypothetical protein
relE	mRNA interferase toxin RelE
group_1424	phage repressor
group_1423	hypothetical protein
csrA_1	Translational regulator CsrA
group_1417	hypothetical protein
csrA_2	Translational regulator CsrA
group_1405	hypothetical protein
group_1404	hypothetical protein
group_1401	hypothetical protein
group_1400	hypothetical protein
group_1396	IS5 family transposase ISMasp6
group_1394	hypothetical protein
legL2_2	leucine-rich repeat-containing protein
higB1	Toxin HigB-1
group_1391	hypothetical protein
group_1385	hypothetical protein
group_1384	hypothetical protein
group_1383	hypothetical protein
group_1381	hypothetical protein
group_1375	hypothetical protein

group_1374	hypothetical protein
group_1371	hypothetical protein
group_1362	hypothetical protein
group_1361	HipA (persistence to inhibition of murein or DNA biosynthesis)
group_1360	hypothetical protein
group_1357	hypothetical protein
group_1353	oxidase
group_1352	hypothetical protein
group_1343	hypothetical protein
group_1342	hypothetical protein
group_1333	hypothetical protein
group_1331	hypothetical protein
group_1330	TraK
group_1329	TraK
group_1323	hypothetical protein
group_1318	putative protein
group_1307	hypothetical protein
group_1306	hypothetical protein
group_1305	hypothetical protein
group_1303	hypothetical protein
group_1302	hypothetical protein
group_1298	hypothetical protein
traJ_2	Protein TraJ
group_1294	hypothetical protein
group_1292	hypothetical protein
secD_1	Protein translocase subunit SecD
group_1289	hypothetical protein
group_1288	TraK

group_1279	hypothetical protein
group_1278	TraK
group_1271	inner membrane protein
group_1270	hypothetical protein
group_1262	hypothetical protein
group_1259	hypothetical protein
group_1253	hypothetical protein
group_1252	hypothetical protein
group_1244	hypothetical protein
lvrB_2	LvrB
insK	Putative transposase InsK for insertion sequence element IS150
group_1236	hypothetical protein
group_1235	hypothetical protein
group_1231	hypothetical protein
group_1222	hypothetical protein
group_1218	putative exported protein
group_1209	single strand DNA binding protein
group_1205	Acetyltransferase
group_1202	hypothetical protein
group_1201	hypothetical protein
group_1199	hypothetical protein
group_1198	hypothetical protein
group_1192	hypothetical protein
group_1185	hypothetical protein
group_1180	hypothetical protein
group_1175	hypothetical protein
group_1171	hypothetical protein
group_1170	hypothetical protein

group_1166	sensory box histidine kinase/response regulator
traM	Protein TraM
group_1154	outer membrane lipoprotein
group_1151	hypothetical protein
group_1132	ISSod6 transposase IS1301
socA	Antitoxin SocA
rayT	REP-associated tyrosine transposase
group_1125	transposase (IS652)
group_1121	hypothetical protein
group_1120	hypothetical protein
group_1119	hypothetical protein
group_1118	hypothetical protein
group_1112	hypothetical protein
group_1102	hypothetical protein
group_1099	antirestriction protein
group_1098	hypothetical protein
group_1094	hypothetical protein
group_1091	Mycothiol acetyltransferase
group_1088	polypeptide deformylase
group_1087	hypothetical protein
group_1086	phage related integrase
group_1068	aminoglycoside N(6')acetyltransferase
group_1059	hypothetical protein
group_1052	hypothetical protein
cas6f	CRISPR-associated endonuclease Cas6f/Csy4
group_1038	hypothetical protein
group_1033	transcriptional regulator TetR family
group_1032	peptidyl-prolyl cis-trans isomerase (rotamase)

group_1029	hypothetical protein
group_1022	hypothetical protein
group_1015	hypothetical protein
group_1011	hypothetical protein
group_1009	hypothetical protein
group_1005	hypothetical protein
group_1004	hypothetical protein
group_1001	hypothetical protein
group_988	hypothetical protein
group_983	hypothetical protein
group_982	alkaline phosphatase
group_978	hypothetical protein
group_974	hypothetical protein
group_972	sensory box (GGDEF/EAL domain)
group_969	hypothetical protein
group_968	hypothetical protein
group_967	hypothetical protein
group_966	2-methoxy-6-polyprenyl-14-benzoquinol methylase mitochondrial
rlmD	23S rRNA (uracil(1939)-C(5))-methyltransferase RlmD
group_963	hypothetical protein
group_962	putative HTH-type transcriptional regulator
group_952	hypothetical protein
glaR	HTH-type transcriptional repressor GlaR
group_947	transposase TnpA
group_945	hypothetical protein
group_942	hypothetical protein
group_933	phage related integrase
group_927	hypothetical protein

group_926	beta-phosphoglucomutase
group_921	hypothetical protein
group_918	hypothetical protein
group_917	hypothetical prophage repressor CI-like protein
group_892	hypothetical protein
lvrA_4	LvrA
group_889	ABC transporter ATP binding protein
group_886	hypothetical protein
group_882	hypothetical protein
traL_2	Protein TraL
group_876	hypothetical; probable transcriptional regulator-like protein
group_875	hypothetical protein
group_869	hypothetical protein
group_868	hypothetical protein
group_862	hypothetical protein
dnaJ_2	Chaperone protein DnaJ
group_861	acetyoacetyl CoA reductase
group_860	hypothetical protein
group_859	hypothetical protein
phaB_4	acetoacetyl CoA reductase
group_855	hypothetical protein
group_850	hypothetical protein
group_846	hypothetical protein
group_842	hypothetical protein
fabI_2	enoyl reductase
group_833	queuosine precursor transporter
group_825	hypothetical protein
group_818	hypothetical protein

group_817	hypothetical protein
group_805	hypothetical protein
group_789	hypothetical protein
group_784	phenazine biosynthesis PhzF
group_783	hypothetical protein
group_777	hypothetical protein
group_770	hypothetical protein
group_766	transcription regulator protein response regulator containing CheY-like receiver domain and HTH DNA-binding domain
group_762	putative exported protein
group_761	hypothetical protein
group_754	UDP-N-acetylglucosamine acyltransferase acyl-[acyl carrier protein]-UDP-N-acetylglucosamine-O-acyltransferase
group_753	hypothetical protein
group_751	transmembrane protein
group_750	LvrA
group_748	lipid A lauroyl acyltransferase
group_743	WaaM
group_742	IS701 family transposase ISAeme11
group_738	ISxcC1 transposase
group_730	hypothetical protein
rfbA_2	polysaccharide ABC transporter permease protein
group_726	hypothetical protein
hipA_1	Serine/threonine-protein kinase toxin HipA
group_716	putative secreted protein
group_715	hypothetical protein
group_710	hypothetical protein
group_709	LvrA
group_704	hypothetical protein

group_699	hypothetical protein
group_698	hypothetical protein
group_690	glycosyl transferase
group_688	hypothetical protein
group_687	putative integrase
group_684	putative integrase
lvrA_5	LvrA
group_677	hypothetical protein
group_673	transcriptional regulator LysR family
group_669	hypothetical protein
group_668	hypothetical protein
group_664	hypothetical protein
glaH	Glutarate 2-hydroxylase
uspE_1	Universal stress protein E
group_643	hypothetical protein
uspE_2	Universal stress protein E
group_641	hypothetical protein
csy2	CRISPR-associated protein Csy2
group_639	hypothetical protein
group_637	hypothetical protein
group_636	transcriptional regulator LysR family
group_635	transposase IS4 family Tn5
group_632	hypothetical protein
group_619	NAD dependent epimerase/dehydratase UDP-glucose-4-epimerase
group_617	hypothetical protein
group_613	hypothetical protein
virB11_2	LvhB11
cas1	CRISPR-associated endonuclease Cas1

lvrA_2	LvrA
group_598	hypothetical protein
group_597	transposase (ISmav2)
group_593	major outer membrane protein
group_592	major outer membrane protein
group_574	hypothetical protein
doeB	N-alpha-acetyl-L-24-diaminobutyric acid deacetylase
group_570	O-antigen initiating glycosyl transferase group 4-UDP-N-acetylmuramyl pentapeptide phosphotransferase/(N-acetylgalactosaminyl transferase TrsF)
group_569	hypothetical protein
group_558	glycosyltransferase
group_556	sulfurylase ThiF
csy3	CRISPR-associated protein Csy3
group_550	hypothetical protein
moaA	GTP 3'8-cyclase
lpxD_2	UDP-3-O-[3-hydroxymyristoyl] glucosamine N-acyltransferase
group_547	hypothetical protein
group_546	transposase (ISSod13)
group_545	hypothetical protein
group_544	hypothetical protein
group_543	hypothetical protein
group_536	hypothetical protein
tdh_2	threonine(-3-)dehydrogenase
group_535	hypothetical protein
group_527	IS630 family transposase ISSpu23
group_525	hypothetical protein
group_519	hypothetical protein
group_513	hypothetical protein
group_507	hypothetical protein

lag1	O-acetyltransferase
recB	RecBCD enzyme subunit RecB
aatA_2	aspartate aminotransferase A
group_494	hypothetical protein
group_493	spore coat polysaccharide biosynthesis protein E (NeuB)
group_489	hypothetical protein
group_485	hypothetical protein
group_481	hypothetical protein
group_480	hypothetical protein
group_474	hypothetical protein
group_471	hypothetical protein
group_470	hypothetical protein
recD2	ATP-dependent RecD-like DNA helicase
ybdK_1	Putative glutamate--cysteine ligase 2
group_468	chemiosmotic efflux system B protein B
group_462	lipid A-disaccharide synthase
group_461	hypothetical protein
group_455	hypothetical protein
group_448	hypothetical protein
nhaA	Na(+)/H(+) antiporter NhaA
group_442	hypothetical protein
ackA2_2	acetate kinase
yfcJ	putative MFS-type transporter YfcJ
group_436	hypothetical protein
group_435	hypothetical protein
group_424	putative protein
group_414	hypothetical protein
group_412	hypothetical protein

int_3	integrase phage related
macA	Macrolide export protein MacA
group_400	MFS transporter
xerC	Tyrosine recombinase XerC
group_390	site specific recombinase
group_389	hypothetical protein
int_1	integrase phage related
group_384	phage related integrase
group_382	phage related integrase
int_2	integrase phage related
group_377	cobalt/zinc/cadmium efflux RND transporter outer membrane protein
group_376	chemiosmotic efflux system B protein C
traA	conjugal transfer protein TraA
group_369	hypothetical protein
group_368	SdbC
group_364	EbhA protein
group_363	hypothetical protein
group_356	cation efflux system HelB
group_351	exported membrane protein
group_347	hypothetical protein
csy1	CRISPR-associated protein Csy1
group_341	hypothetical protein
group_324	hypothetical protein
group_319	proline/betaine transporter ProP6
group_317	major facilitator family transporter
group_306	hypothetical protein
group_291	hypothetical protein
group_290	hypothetical protein

group_287	hypothetical protein
iucD	L-lysine N6-monooxygenase
group_263	metallo-beta lactamase family protein
group_259	membrane protein Tfp pilus assembly pilus retraction ATPase PilT
group_249	hypothetical protein
pleD_1	sensor histidine kinase
group_247	putative phage protein
group_246	hypothetical protein
group_243	hypothetical protein
group_239	putative protein kinase
group_231	hypothetical protein
group_227	phosphate acetyl/butyryltransferase family protein) includes: (de)hydratase mit MaoC domain)
group_220	hypothetical protein
group_216	polysaccharide ABC transporter ATP binding protein
group_211	hypothetical protein
group_202	hypothetical protein
group_201	proton/peptide symporter family protein
group_185	hypothetical protein
group_182	hypothetical protein
group_181	thymidine phosphorylase TdRPase
group_178	hypothetical protein
murA_1	UDP-N-acetylglucosamine 1-carboxyvinyltransferase
legA10_2	ankyrin repeat-containing protein
treA	Periplasmic trehalase
group_172	hypothetical protein
group_170	membrane protein
group_167	hypothetical protein
group_160	hypothetical protein

group_159	hypothetical protein
group_155	hypothetical protein
group_148	virulence factor MviN
group_139	hypothetical protein
group_133	hypothetical protein
group_128	glutathione-regulated potassium efflux system
group_127	glutathione-regulated potassium efflux system
group_126	hypothetical protein
group_116	hypothetical protein
group_115	hypothetical protein
group_114	inner membrane protein
group_112	inner membrane protein
group_111	hypothetical protein
group_110	inner membrane protein
group_108	hypothetical protein
phbC_2	polyhydroxyalkanoic synthase
group_102	hypothetical protein
group_101	hypothetical protein
group_99	hypothetical protein
group_90	hypothetical protein
group_88	conjugative coupling factor TraD
traI_2	Protein TraI
group_85	Kup system potassium uptake protein
virD4_2	LvhD4
group_83	hypothetical protein
group_80	hypothetical protein
group_79	hypothetical protein
group_77	O-antigen acetylase

arcB	Aerobic respiration control sensor protein ArcB
group_68	hypothetical protein
cadA1	cadmium translocating P-type ATPase CadA
group_62	hypothetical protein
group_61	hypothetical protein
group_60	copper efflux ATPase
group_56	hypothetical protein
group_50	hypothetical protein
group_45	hypothetical protein
group_37	hypothetical protein
pacL_1	cation efflux transporter
mgtA	cation efflux transporter
group_31	hypothetical protein
pacL_2	cation efflux transporter
group_29	Type IV secretory protein VirB4 components
group_27	hypothetical protein
group_26	hypothetical protein
hsdR	Type-1 restriction enzyme R protein
group_23	hypothetical protein
group_21	hypothetical protein
group_20	chemiosmotic efflux system B protein A
group_17	cobalt/zinc/cadmium efflux RND transporter permease protein HelA
group_13	hypothetical protein
group_11	hypothetical protein
cas3	CRISPR-associated nuclease/helicase Cas3 subtype I-F/YPEST
group_10	hypothetical protein
group_6	hypothetical protein
group_4	Sid related protein-like protein

group_3	hypothetical protein
group_1	hypothetical protein
rhlE	ATP-dependent RNA helicase RhlE
dltA	D-alanine--D-alanyl carrier protein ligase
group_1502	hypothetical protein
group_1502	hypothetical protein
group_1395	hypothetical protein
group_741	hypothetical protein
group_1653	IS630 family transposase ISSpu23

Table S5: Recombinant region identified in SPF635 and SPF636

**Genome position**	**Gene ID**	**Gene name**	**product**
907470..907991	MNEPOAHH_00806		rhamboid family protein
907988..908890	MNEPOAHH_00807		peptidase M23/M37 family
908899..910230	MNEPOAHH_00808		exonuclease VII large subunit
910380..912104	MNEPOAHH_00809	tolC	agglutination protein
912055..912789	MNEPOAHH_00810		periplasmic protein
912805..914724	MNEPOAHH_00811		two component histidine kinase GGDEF domain protein
914860..915585	MNEPOAHH_00812		hypothetical protein
915563..916903	MNEPOAHH_00813		flavin containing monooxygene
917044..917412	MNEPOAHH_00814		hypothetical protein
917378..918187	MNEPOAHH_00815		IS4 family transposase ISRm16
918393..919238	MNEPOAHH_00816		oxidoreductase dehydrogenase short chain
919415..920701	MNEPOAHH_00817	wbpA	UDP-N-acetyl-D-glucosamine 6-dehydrogenase

Table S7: Summary of functional annotation of plasmid sequence

locus_tag	length_bp	gene	EC_number	COG	product
ORF1	1050				hypothetical protein
ORF2	843				hypothetical protein
ORF3	1314				hypothetical protein
ORF4	1215				hypothetical protein
ORF5	210				hypothetical protein
ORF6	291				hypothetical protein

ORF7	2049	arcB	2.7.13.3	COG0642	Aerobic respiration control sensor protein ArcB
ORF8	732	yvmC	2.3.2.22		Cyclo(L-leucyl-L-leucyl) synthase
ORF9	165				hypothetical protein
ORF10	276				hypothetical protein
ORF11	2232	copA	7.2.2.8	COG2217	Copper-exporting P-type ATPase
ORF12	1299	czcC_1			Cobalt-zinc-cadmium resistance protein CzcC
ORF13	969	mdtA			Multidrug resistance protein MdtA
ORF14	3207	czcA_1			Cobalt-zinc-cadmium resistance protein CzcA
ORF15	456				hypothetical protein
ORF16	1458	mmcO	1.16.3.1	COG2132	Multicopper oxidase MmcO
ORF17	330	yhjQ		COG1145	putative cysteine-rich protein YhjQ
ORF18	372				hypothetical protein
ORF19	162				hypothetical protein
ORF20	1494				hypothetical protein
ORF21	135				hypothetical protein
ORF22	780				hypothetical protein
ORF23	192				hypothetical protein
ORF24	456				hypothetical protein

ORF25	735	artI		COG0834	Putative ABC transporter arginine-binding protein 2
ORF26	1248	czcC_2			Cobalt-zinc-cadmium resistance protein CzcC
ORF27	1239				hypothetical protein
ORF28	3150	czcA_2			Cobalt-zinc-cadmium resistance protein CzcA
ORF29	297	nmtR			HTH-type transcriptional regulator NmtR
ORF30	2151	cadA	3.6.3.3		Cadmium-transporting ATPase
ORF31	330				hypothetical protein
ORF32	375				hypothetical protein
ORF33	165				hypothetical protein
ORF34	906	czcD			Metal cation efflux system protein CzcD
ORF35	744	phbB	1.1.1.36		acetoacetyl-CoA reductase
ORF36	210	capB			Cold shock protein CapB
ORF37	135				hypothetical protein
ORF38	432				hypothetical protein
ORF39	1035	xerC			Tyrosine recombinase XerC
ORF40	255				hypothetical protein
ORF41	297	parE1		COG3668	Toxin ParE1
ORF42	195				hypothetical protein

ORF43	270				hypothetical protein
ORF44	627				hypothetical protein
ORF45	357				hypothetical protein
ORF46	381	mazG	3.6.1.8	COG1694	Nucleoside triphosphate pyrophosphohydrolase
ORF47	144				hypothetical protein
ORF48	822				hypothetical protein
ORF49	303				hypothetical protein
ORF50	306				hypothetical protein
ORF51	420	klcA			Antirestriction protein KlcA
ORF52	132				hypothetical protein
ORF53	201				hypothetical protein
ORF54	330				hypothetical protein
ORF55	291				hypothetical protein
ORF56	804				hypothetical protein
ORF57	264				hypothetical protein
ORF58	702				hypothetical protein
ORF59	210				hypothetical protein
ORF60	303				hypothetical protein

ORF61	369				hypothetical protein
ORF62	423				hypothetical protein
ORF63	5835	traI			conjugative transfer relaxase/helicase TraI
ORF64	1854	traD			type IV conjugative transfer system coupling protein TraD
ORF65	624				hypothetical protein
ORF66	2892				conjugal transfer protein TraG
ORF67	1383				conjugal transfer protein TraH
ORF68	495	trbB			type-F conjugative transfer system pilin assembly thiol-disulfide isomerase TrbB
ORF69	783	traF			type-F conjugative transfer system pilin assembly protein TraF
ORF70	1812	traN			type-F conjugative transfer system mating-pair stabilization protein TraN
ORF71	666	trbC			type-F conjugative transfer system pilin assembly protein TrbC
ORF72	978				conjugal transfer protein TraU
ORF73	636	traW			type-F conjugative transfer system protein TraW
ORF74	366				pilus assembly protein
ORF75	2550	traC			type IV secretion system protein TraC
ORF76	228				type IV conjugative transfer system protein TraV
ORF77	1467				conjugal transfer protein TraB
ORF78	735	traK			type-F conjugative transfer system secretin TraK

ORF79	561				conjugal transfer protein TraE
ORF80	291	traL			type IV conjugative transfer system protein TraL
ORF81	312				conjugal transfer protein TraA
ORF82	372				hypothetical protein
ORF83	402				hypothetical protein
ORF84	753				conjugal transfer protein TraT
ORF85	225	csrA			carbon storage regulator CsrA
ORF86	201				hypothetical protein
ORF87	522				hypothetical protein
ORF88	999				Vir protein
ORF89	651				hypothetical protein
ORF90	900				alpha/beta hydrolase
ORF91	1092				amino acid ABC transporter substrate-binding protein
ORF92	2085				HAMP domain-containing histidine kinase
ORF93	225				hypothetical protein
ORF94	969				response regulator
ORF95	429				hypothetical protein

Table S8: Primer candidates for ST2858 screening. Gene annotation was based on Prokka annotation of SPF580.

Gene	Primer	Melting temp (°C)	Product size (bp)
783	Forward - ATGGGAAGATGATATACCGACCG<br>Reverse - GTGCTTCAGTGATTAAGGCGTC	64-65	278
784	Forward - TAATTCAACCACGAACAGTTGC<br>Reverse – AAAATCTAGTGAGGGATGTGAAGC	62-63	525
786	Forward - ATGGATCTTACAACTGTTTTAATAGC<br>Reverse – GATAATTCTCTCTATGAAATCATTAGC	58-59	543
787	Forward - ATGTCTGAGGAAGCACTGAGAAT<br>Reverse – CTGGCTGGGAACTAACGCTAAT	62-64	424
1045	Forward - TTGCAATTGGACATATTGTAGG<br>Reverse – CATAATAAAGAAACAATATAACTTGTAATCC	64	418
3056	Forward - GTGCCGAAACATGATGTTATCC<br>Reverse - TCAATTACCATAACGACCGCAT	59-60	252

Table S9: Summary of regions of the 675 isolates or DNA extraction that were collected in Quebec Canada from 2021-2022 and screened with ST2858 specific primers. Those from health facility A (HF-A) within Montreal are highlighted.

Region	Isolates	DNA extractions
Unknown	87	
Montréal<br>(from HF-A)	393<br>(187)	49<br>(11)
Lanaudière	2	
Centre-du-Québec	7	
Laval	11	3
Saguenay--Lac-Saint-Jean	11	
Chaudière-Appalaches	16	
Montérégie	48	
Estrie	11	
Outaouais	3	
Abitibi-Témiscamingue	6	
Mauricie	1	
Capitale-Nationale	3	
Grand total	599	52
